# Insights Into the Function of the NuA4 Complex in Plants

**DOI:** 10.3389/fpls.2020.00125

**Published:** 2020-02-21

**Authors:** Loreto Espinosa-Cores, Laura Bouza-Morcillo, Javier Barrero-Gil, Verónica Jiménez-Suárez, Ana Lázaro, Raquel Piqueras, José A. Jarillo, Manuel Piñeiro

**Affiliations:** Centro de Biotecnología y Genómica de Plantas, Universidad Politécnica de Madrid (UPM) - Instituto Nacional de Investigación y Tecnología Agraria y Alimentaria (INIA), Madrid, Spain

**Keywords:** chromatin, histone acetylation, NuA4, TIP60, SWR1, Arabidopsis, development, flowering time

## Abstract

Chromatin remodeling plays a key role in the establishment and maintenance of gene expression patterns essential for plant development and responses to environmental factors. Post-translational modification of histones, including acetylation, is one of the most relevant chromatin remodeling mechanisms that operate in eukaryotic cells. Histone acetylation is an evolutionarily conserved chromatin signature commonly associated with transcriptional activation. Histone acetylation levels are tightly regulated through the antagonistic activity of histone acetyltransferases (HATs) and histone deacetylases (HDACs). In plants, different families of HATs are present, including the MYST family, which comprises homologs of the catalytic subunit of the Nucleosome Acetyltransferase of H4 (NuA4) complex in yeast. This complex mediates acetylation of histones H4, H2A, and H2A.Z, and is involved in transcriptional regulation, heterochromatin silencing, cell cycle progression, and DNA repair in yeast. In Arabidopsis and, other plant species, homologs for most of the yeast NuA4 subunits are present and although the existence of this complex has not been demonstrated yet, compelling evidence supports the notion that this type of HAT complex functions from mosses to angiosperms. Recent proteomic studies show that several Arabidopsis homologs of NuA4 components, including the assembly platform proteins and the catalytic subunit, are associated *in vivo* with additional members of this complex suggesting that a NuA4-like HAT complex is present in plants. Furthermore, the functional characterization of some Arabidopsis NuA4 subunits has uncovered the involvement of these proteins in the regulation of different plant biological processes. Interestingly, for most of the mutant plants deficient in subunits of this complex characterized so far, conspicuous defects in flowering time are observed, suggesting a role for NuA4 in the control of this plant developmental program. Moreover, the participation of Arabidopsis NuA4 homologs in other developmental processes, such as gametophyte development, as well as in cell proliferation and stress and hormone responses, has also been reported. In this review, we summarize the current state of knowledge on plant putative NuA4 subunits and discuss the latest progress concerning the function of this chromatin modifying complex.

## Introduction

In eukaryotic organisms, DNA is present in the nucleus in a highly compacted structure known as chromatin, in which nucleosomes are the basic structural units. Each nucleosome encompasses a histone octamer (two H2A-H2B dimers and an H3-H4 tetramer), and 147 bp of DNA wrapped around the histone octamer (Luger et al., 1997). However, the fact that DNA is packaged into the nucleus complicates the access of nuclear machinery that mediates different cellular processes such as transcription, replication or DNA repair (Luger et al., 2012). Chromatin needs to be relaxed by remodelers to allow these processes to take place, making the structure of nucleosomes very dynamic (Venkatesh and Workman, 2015). In particular, the reorganization of chromatin is pivotal for the establishment of gene expression patterns that drive developmental programs and environmental responses in eukaryotes (Xiao et al., 2017). Chromatin remodeling can be carried out by complexes that i) use ATP hydrolysis to alter the interaction between the DNA and the histone octamer non-covalently (Clapier et al., 2017); ii) catalyze the exchange of canonical histones by histone variants, which have specialized functions and differ in sequence from the canonical histones (Talbert and Henikoff, 2017); and iii) are involved in the covalent modification of histones and DNA, which affect the condensation status of chromatin (Bhaumik et al., 2007). The post-translational modification (PTM) of histones occurs mostly in the amino-terminal tails, and is one of the most important mechanisms to regulate chromatin dynamics. These covalent modifications include, among others, lysine (K) acetylation, ubiquitination, and methylation, arginine methylation, and phosphorylation (Bannister and Kouzarides, 2011). In addition, DNA is also a methylation target (Law and Jacobsen, 2010). Many of these modifications have functions in transcription, and some may also accomplish roles in DNA repair, replication or chromatin condensation (Kouzarides, 2007).

The combination and crosstalk among different PTMs constitutes a histone code that sets the basis for epigenetic transcriptional regulation and adds an extra level of regulation overlying those mediated by transcription factors (Strahl and Allis, 2000; Rothbart and Strahl, 2014). Some histone modifications such as trimethylation of lysine 27 in histone H3 (H3K27me3) and H3K9me2 are commonly associated with transcriptional repression (Shahbazian and Grunstein, 2007; Schuettengruber et al., 2017), whereas H3 and H4 acetylation, H3K4me3, and H3K36me3 are marks linked to transcriptionally active states (Pokholok et al., 2005; Black et al., 2012). PTMs act as recruitment platforms for effector proteins that modify the transcriptional status of underlying genes. However, histone acetylation has an additional physical effect on chromatin structure. The addition of a negatively charged acetyl group to K has been proposed to neutralize the positive charge of K in histones. This reduces the affinity of the histone tail for the DNA and contributes to release the interaction between histones and DNA, and to open the chromatin (Barnes et al., 2019). Due to its great impact on a myriad of cellular and developmental processes, the antagonistic action of two classes of enzymes, histone deacetylases (HDACs), and histone acetyltransferases (HATs), has been the subject of a growing number of studies (Liu et al., 2014; Hu et al., 2019).

HATs are evolutionarily conserved from yeast to humans, including plants, and they usually function as multiprotein complexes defined by the catalytic subunit responsible for the transfer of acetyl groups to K residues (Lee and Workman, 2007). There are at least four families of HATs including GNAT (Gcn5-related N-acetyltransferase), MYST (MOZ, YbF2, Sas2, Tip60-like), p300/CREB-binding protein (CBP), and TAF_II_250 families. Eukaryotic genomes usually contain multiple members of each family illustrating their relevance for chromatin function. For example, in *Arabidopsis thaliana* twelve HATs have been identified. Three of them belong to the GNAT-MYST superfamily, five to the p300/CBP family, two to the TAF_II_250, and two more to the MYST family (Berr et al., 2011; Liu et al., 2016).

The MYST family of HATs is the largest one and is present in all eukaryotes. One of the best characterized complexes included in this family is the yeast Nucleosome Acetyltransferase of Histone H4 (NuA4) complex, highly conserved in eukaryotes. NuA4 is involved in different genomic processes such as DNA damage repair and transcription, heterochromatin silencing, cell cycle progression, and chromosome stability (Clarke et al., 1999; Doyon and Côté, 2004; Lee and Workman, 2007; Lu et al., 2009; Uprety et al., 2012; Bruzzone et al., 2018; Hodges et al., 2019). NuA4 was initially described in yeast to acetylate nucleosomal histones at specific gene promoters (Ginsburg et al., 2009), although later studies showed that this complex is also present in actively transcribed coding sequences (Steunou et al., 2016). Besides acetylating histone H4, NuA4 also acts on histone H2A (Boudreault et al., 2003) and the variant H2A.Z (Millar et al., 2006; Valdés-Mora et al., 2012). In mammals and flies, NuA4 evolved into a hybrid complex known as TIP60 (Cai et al., 2003), formed by subunits that in yeast belong to NuA4 and the ATP-dependent SWI2/SNF2-Related 1 chromatin remodeling complex (SWR1), that mediates the exchange of histone H2A by the histone variant H2A.Z (Voss and Thomas, 2009). NuA4 also acetylates non-histone protein substrates in yeast and metazoans, ascribing to this complex additional roles in controlling metabolism, autophagy, and homeostasis (Lin et al., 2009; Narita et al., 2019).

Most of the yeast NuA4 subunits are widely conserved in plants where more than one homolog for members of the complex are frequently found. This is the case of *Arabidopsis thaliana,* in which many NuA4 components, including the putative catalytic subunits HISTONE ACETYLTRANSFERASE OF THE MYST FAMILY (HAM1/2) (Latrasse et al., 2008), are duplicated. Recent proteomic analyses performed with Arabidopsis homologs of NuA4 subunits have revealed that most of the components of this complex associate to each other *in vivo* (Bieluszewski et al., 2015; Tan et al., 2018; Crevillén et al., 2019). In plants, mutants for different NuA4 subunits characterized so far display pleiotropic vegetative and reproductive alterations, such as abnormal flowering time (Latrasse et al., 2008; Umezawa et al., 2013; Xiao et al., 2013; Bu et al., 2014; Xu et al., 2014; Bieluszewski et al., 2015; Gómez-Zambrano et al., 2018; Peng et al., 2018; Crevillén et al., 2019), suggesting the involvement of NuA4 in the control of central plant developmental processes through acetylation-mediated regulation of gene expression. In this review, we discuss the possible existence of NuA4 in plants, and describe the biological functions carried out by different homolog subunits of NuA4 studied in Arabidopsis. We speculate with future directions of the research aimed at confirming the conservation of this HAT complex in plants and characterizing its role in the control of gene expression to regulate different plant developmental programs and environmental responses.

## Organization Of The NuA4 Complex: Lessons From Yeast And Metazoans

The structure and molecular architecture of yeast NuA4 have been characterized by cryo-electron microscopy as well as by studying the interactions between the different subunits (Chittuluru et al., 2011; Wang et al., 2018a). Yeast NuA4 comprises 13 subunits including the catalytic subunit Esa1, together with Enhancer of polycomb-like1 (Epl1), the inhibitor of growth (ING) factor Yng2, Esa1-associated factor 6 (Eaf6), Transcription-associated protein 1 (Tra1), Eaf1, Eaf3, Eaf5, Eaf7, Actin-related protein 4 (Arp4), Actin1 (Act1), Yeast all fused gene from chromosome 9 (Yaf9), and SWR1 COMPLEX 4 (Swc4)/Eaf2 subunits (**Table 1**). Interestingly, most of the components of this 1.0-MDa complex are shared with other chromatin remodeling complexes. For instance, Swc4, Yaf9, Arp4, and Act1 are members of SWR1, and therefore are also involved in the exchange of H2A-H2B by H2A.Z-H2B dimers (Gerhold and Gasser, 2014). Tra1 is also part of the recruitment module in SAGA and SAGA-like (SLIK)/SALSA complexes (Helmlinger and Tora, 2017). Arp4 and Act1 are additionally present in the INO80 ATP-dependent chromatin remodeling complex (Chen et al., 2013), while Eaf3 is a component of the Reduced Potassium Dependency-3 Small (Rpd3S) HDAC complex (Carrozza et al., 2005; Keogh et al., 2005), and Eaf6 is also found in the yeast NuA3 (Taverna et al., 2006), HUMAN ACETYLASE BINDING TO ORC1 (HBO1) (Avvakumov et al., 2012), and MONOCYTIC LEUKEMIC ZINC-FINGER PROTEIN (MOZ)/MOZ RELATED FACTOR (MORF) HAT complexes (Yang and Ullah, 2007). Even the catalytic subunit in metazoans complexes is not exclusively present in TIP60 since it can also be found in HBO1 and MOZ HATs (Xu et al., 2016). Highlighting the strong conservation of this complex throughout evolution, most of the NuA4 subunits display high homology with the TIP60 HAT complex in *Homo sapiens*, in which twelve out of the thirteen subunits are conserved (**Table 1**) (Cai et al., 2003; Doyon and Côté, 2004).

**Table 1:** NuA4 conserved subunits from yeast to humans.

*NuA4 conserved subunits*					
	*S. cerevisiae*	*A. thaliana*	*O. sativa*	*D. melanogaster* (TIP60)	*H. sapiens* (TIP60)
**Piccolo NuA4**	Epl1	AtEPL1A (AT1G16690) AtEPL1B (AT1G79020)	Os09g0284600 Os08g0338900	E(Pc)	EPC1
	Eaf6	AtEAF6 (AT4G14385)	Os12g0298600 Os01g0233400	Eaf6	EAF6
	Yng2	AtING1 (AT3G24010) AtING2 (AT1G54390)	Os03g0143600 Os03g0748200	ING3	ING3
	Esa1	AtHAM1 (AT5G64610) AtHAM2 (AT5G09740)	Os07g0626600	TIP60	TIP60
**Assembly platform**	Tra1	AtTRA1 (AT4G36080) AtTRA2 (AT2G17930)	Os07g0645100	dTRA1	TRRAP
	Eaf1	AtEAF1A (AT3G24880) AEAF1B (AT3G24870)	Os08g0177300	Domino/p400	Domino/p400
**TINTIN**	Eaf5	-			
	Eaf7	AtEAF7 (AT1G26470)	Os05g0512500	MRGBP	MRGBP
	Eaf3	AtMRG1 (AT4G37280) AtMRG2 (AT1G02740)	OsMRG701 (Os04g0101300) OsMRG702 (Os11g0545600)	MRG15	MRG15
**SWR1 shared module**	Swc4	AtSWC4 (AT2G47210)	Os05g0540800	DMAP1	DMAP1
	Yaf9	AtYAF9A (AT5G45600) AtYAF9B (AT2G18000)	Os06g0137300	GAS41	GAS41
	Act1	8, including AT2G37620 and AT3G53750	Os03g0718150	Act88F	Act1
	Arp4	AtARP4 (AT1G18450) AtARP4A (AT1G73910)	Os08g0137200	BAP55	BAF53

Composition of NuA4-like complexes from model organisms such as S. cerevisiae, D. melanogaster, H. sapiens (Doyon and Côté, 2004; Sapountzi et al., 2006), A thaliana and O sativa are included. Annotations for Arabidopsis and rice NuA4 homolog proteins come from the Arabidopsis Information Resource (TAIR) (https://www.arabidopsis.org/) and the Rice Annotation Project Database (https://rapdb.dna.affrc.go.jp/index.html), respectively.

Interestingly, the biochemical isolation of two NuA4 subcomplexes lacking the full array of subunits has been reported in yeast: the Piccolo (Boudreault et al., 2003), and the Trimer Independent of NuA4 involved in Transcription Interactions with Nucleosomes (TINTIN) (Rossetto et al., 2014; Wang et al., 2018a). Both subcomplexes work independently of the core NuA4 and have specific functions (Mitchell et al., 2008). A recent study has addressed the spatial structure of NuA4 by performing single-particle electron microscopy. This approach has revealed that NuA4 has a trilobal structure with a central core and two lobes. The central core contains Tra1, the Piccolo module and Eaf1, while lobe 1 is formed by the TINTIN sub-module, and lobe 2 is composed of the four subunits shared with SWR1 (Setiaputra et al., 2018). Studies carried out in parallel performed cryoelectron microscopy and released the crystal structure of the Piccolo NuA4 core complex unveiling the histone H4 acetylation mechanism in the context of the nucleosome (Xu et al., 2016). This type of approach is shedding light on how this multisubunit complex assembles with the nucleosomal substrate to regulate gene expression.

### A Small Version of NuA4: the Piccolo NuA4 Complex

The yeast Piccolo NuA4 is composed of Esa1, Epl1, Yng2, and Eaf6 subunits (**Table 1**). This complex is responsible for the non-targeted Esa1-mediated acetylation of chromatin, and also for the interaction of NuA4 with the nucleosome core particle (Chittuluru et al., 2011). Esa1 is the only essential acetyltransferase in *Saccharomyces cerevisiae* (Allard et al., 1999). This subunit alone is able to acetylate free histones while it acetylates nucleosomal H4, H2A, and H2A.Z histones *in vivo* when is present in Piccolo NuA4 complex (Keogh et al., 2006; Millar et al., 2006; Lu et al., 2009; Mehta et al., 2010). Esa1 contains a chromodomain (CHD) at the N-terminus and a C-terminal MYST domain. Mutations in the CHD domain, which is required to acetylate nucleosomes *in vitro* but not free histones, are lethal (Doyon and Côté, 2004; Steunou et al., 2016). Esa1 was originally described to be required for double-strand breaks repair (Bird et al., 2002; Lee and Workman, 2007). In addition, as a component of Piccolo NuA4, this subunit has a role in transcriptional regulation of ribosomal protein genes (Uprety et al., 2015) and autophagy response (Yi et al., 2012).

The Epl1 subunit is essential for the interaction of the Piccolo NuA4 complex with nucleosomes (Chittuluru et al., 2011). This is the yeast ortholog of the human EPC1/2 paralogs and the *Drosophila melanogaster* Enhancer of Polycomb (E(Pc)) protein, originally described as an enhancer of trithorax and polycomb mutations (Searle and Pillus, 2018). Epl1 bears two differentiated domains: the C-terminus, which connects Epl1 and the Piccolo complex to the rest of NuA4 through Eaf1, and the EPcA domain in the N-terminus, which physically interacts with the rest of subunits of the Piccolo complex. A short region of the EPcA domain is required for binding to nucleosomes and histone H2A tail, an interaction necessary for the acetylation of nucleosomal H4 (Steunou et al., 2016). The EPcA domain interacts with Esa1 promoting its activation. Then, Esa1 binds the nucleosome through its CHD domain, projecting its catalytic pocket towards the N-terminal tail of H4. The acetylation occurs through a double recognition mechanism of a short sequence of the histone H4 N-terminal tail and the spatial orientation of the histone after the binding of Esa1 with the nucleosome (Xu et al., 2016).

Yng2 is also critical for the Piccolo NuA4 HAT activity on nucleosomes *in vitro* and histone H4 acetylation *in vivo* (Steunou et al., 2016). This subunit belongs to the highly conserved ING tumor suppressor family (Aguissa-Touré et al., 2011) and contains a Plant Homeo-Domain (PHD) and a short polybasic region at its C-terminal domain (Guérillon et al., 2013). The PHD domain binds H3K4me3 near the transcription start sites (TSS) of active genes (Peña et al., 2006). The recognition of H3K4me3 by Yng2 both at the promoter and coding sequences of genes has been proposed to recruit NuA4 to gene promoter regulatory regions. Subsequently, Yng2 positions the Piccolo complex for the acetylation of specific K residues of H4 and H2A histones, providing to this complex the function of maintaining the basal levels of H4 and H2A acetylation (Chittuluru et al., 2011).

The fourth Piccolo subunit, Eaf6, is a small 13-kDa protein without known domains except for a putative leucine zipper region (Doyon and Côté, 2004). The contribution of Eaf6 to the transcriptional regulation mediated by NuA4 has not been fully addressed and awaits further characterization. Recent observations reveal that Eaf6 (in humans, CENP-28) is also present at the centromere, and participates in the induction of centromeric transcription (Molina et al., 2016), possibly acting independently of NuA4.

### The Assembly Platform of NuA4 Contains Eaf1 and Tra1 Subunits

Among the subunits of yeast NuA4, Eaf1 is, together with Epl1, the only subunit present exclusively in this complex (**Table 1**) (Auger et al., 2008; Wang et al., 2018a). Eaf1 directly contacts multiple subunits and occupies the central portion of the NuA4 core. This protein has been proposed to be the assembly platform of NuA4 (Mitchell et al., 2008). In fact, the removal of Eaf1 subunit results in the loss of NuA4 integrity and the collapse of the full complex (Mitchell et al., 2008; Setiaputra et al., 2018). This protein contains a SANT (Swi3, Ada2, N-Cor, and TFIIIB) domain involved in interactions with DNA and histone tails, and also shows structural similarities with p400/Domino, a subunit of TIP60 (**Table 1**) (Cai et al., 2003). Eaf1 directly binds Tra1 through its SANT domain and both proteins constitute the assembly platform of NuA4. Eaf1 also contains a Helicase/SANT-associated (HSA) domain that interacts with the Epl1 C-terminus and bridges the Piccolo module to the rest of NuA4 (Wang et al., 2018a).

Tra1, known as TRRAP in humans, is another important and conserved subunit of the central core of the yeast NuA4 (**Table 1**). There are few demonstrated interactions between this large protein and the rest of NuA4 components, and is located in the opposite domain of the Piccolo complex (Chittuluru et al., 2011). This subunit belongs to the Phosphatidylinositol-3 kinase-related kinase (PIKK) family and is an essential protein since its deletion is lethal in yeast and mammals (Helmlinger et al., 2011; Berg et al., 2018). Recent analysis of the three-dimensional structure of this protein by electron microscopy indicates that Tra1 has a rigid structure, highly conserved in both SAGA and NuA4 complexes (Cheung and Díaz-Santín, 2019).

### The TINTIN Complex

TINTIN is a NuA4 subcomplex composed of Eaf3, Eaf5, and Eaf7 subunits in yeast (**Table 1**) (Cheng and Côté, 2014; Rossetto et al., 2014; Wang et al., 2018a). This complex is tethered to NuA4 through the interaction of Eaf5 with Eaf1. In turn, Eaf7 connects Eaf5 with Eaf3 (Auger et al., 2008; Rossetto et al., 2014). Mutants of these subunits do not show the same phenotypic alterations as the other subunits of the complex, suggesting that TINTIN may have additional functions to those exerted by NuA4 (Mitchell et al., 2008). TINTIN appears more enriched over coding regions than in promoters, suggesting its possible role in transcriptional elongation (Rossetto et al., 2014). Additional TINTIN co-transcriptional roles in mRNA processing, termination, and quality control have been reported revealing some connections with the mRNA splicing machinery and the nuclear exosome (Bhat et al., 2015).

Eaf3 and the human MORF4-related gene on chromosome 15 (MRG15) homolog belong to the Morf Related Gene (MRG) protein family and act as H3K36me3 “readers” (**Table 1**) (Reid et al., 2004). The Eaf3 subunit contains different specific domains: a CHD domain at its N-terminus responsible for binding H3K36 methylated residues, a putative DNA binding region, and a large highly conserved MRG domain. Eaf3 seems to be crucial for proper histone acetylation toward the 3′ end of actively transcribed coding sequences in a process that does not affect the association of the TINTIN complex with these regions (Steunou et al., 2016).

In the Eaf5 protein no functional domains have been identified, and its role does not seem to be critical for NuA4 or TINTIN complexes since the loss-of-function of this protein does not cause abnormal phenotypes in yeast (Mitchell et al., 2008; Rossetto et al., 2014). Moreover, the gene is absent in higher eukaryotes, including plants (Doyon and Côté, 2004; Doyon et al., 2004). Conversely, the third TINTIN subunit, Eaf7, is widely conserved from yeast to humans (**Table 1**) (Sathianathan et al., 2016). In humans, the Eaf7 homolog, MRG/MORF4L-binding protein (MRGBP), forms dimers with MRG15 independently of the TIP60 complex, possibly constituting the human TINTIN subcomplex (Cheng and Côté, 2014; Bhat et al., 2015).

### Accessory NuA4 Subunits Shared With SWR1

Four yeast NuA4 proteins, Yaf9, Swc4, Arp4, and Act1, are also present in the chromatin remodeling complex SWR1 (**Table 1**) (Altaf et al., 2010), suggesting a functional interplay between both remodelers. Indeed, NuA4-dependent acetylation of nucleosomal histones H4 and H2A directly promotes the incorporation of H2A.Z by SWR1 (Altaf et al., 2010). Once incorporated, H2A.Z is also acetylated by NuA4 (Millar et al., 2006; Valdés-Mora et al., 2012). Yaf9 contributes to the functions of both NuA4 and SWR1, and it has been implicated in transcriptional regulation, histone acetylation, DNA repair, chromosome segregation, cellular resistance to microtubule depolymerization, and response to spindle stress (Klein et al., 2018). Yaf9 contains a YEATS (Yaf9, ENL, AF9, Taf14, Sas5) domain, an evolutionarily conserved module that binds histones H3 and H4 *in vitro* (Wang et al., 2009), and that also recognizes H3K27ac in nucleosomes, guiding the replacement of H2A-H2B dimers with H2A.Z-H2B by SWR1 at gene promoters (Klein et al., 2018).

Swc4/Eaf2 contains an N-terminal SANT domain that recognizes both histones and DNA, and a C-terminal Yaf9-interacting domain (Bittner et al., 2004). This subunit is the homolog of the human DNA methyltransferase-associated protein 1 (DMAP1; **Table 1**) (Rountree et al., 2000), suggesting an interplay of Swc4 with the DNA methylation pathways. The fact that Swc4 deletion in yeast did not broadly affect global acetylation levels of histone H4 suggests that it may regulate site-specific roles of NuA4, likely mediating the recruitment of both NuA4 and SWR1 to target genes and coupling acetylation and H2A.Z deposition (Zhou et al., 2010).

The ARP4 protein is a member of the ARP superfamily, a branch of an ancient and highly divergent family of proteins present in all eukaryotes and whose primary sequences display homology to actins (Kandasamy et al., 2005). Nuclear actins (N-actins) control different nucleic acid transitions as part of chromatin remodeling complexes (Olave et al., 2002). Actin and Arp4 form a conjugated pair, and despite being widely conserved in eukaryotes, their structures and roles within the chromatin remodeling complexes have remained obscure until recently (Cao et al., 2016). N-actins and Arp4 are incorporated into different chromatin regulatory complexes through a common motif, the HSA domain (Szerlong et al., 2008). Yeast Arp4 is involved in DNA repair, and it has been suggested to interact with acetylated H4 tails (Bird et al., 2002). Altogether, these observations suggest that the shared subunits between SWR1 and NuA4 may cooperatively enable the association of these complexes with chromatin.

## Growing Evidence For The Presence Of NuA4 In Plants

**Figure 1:**
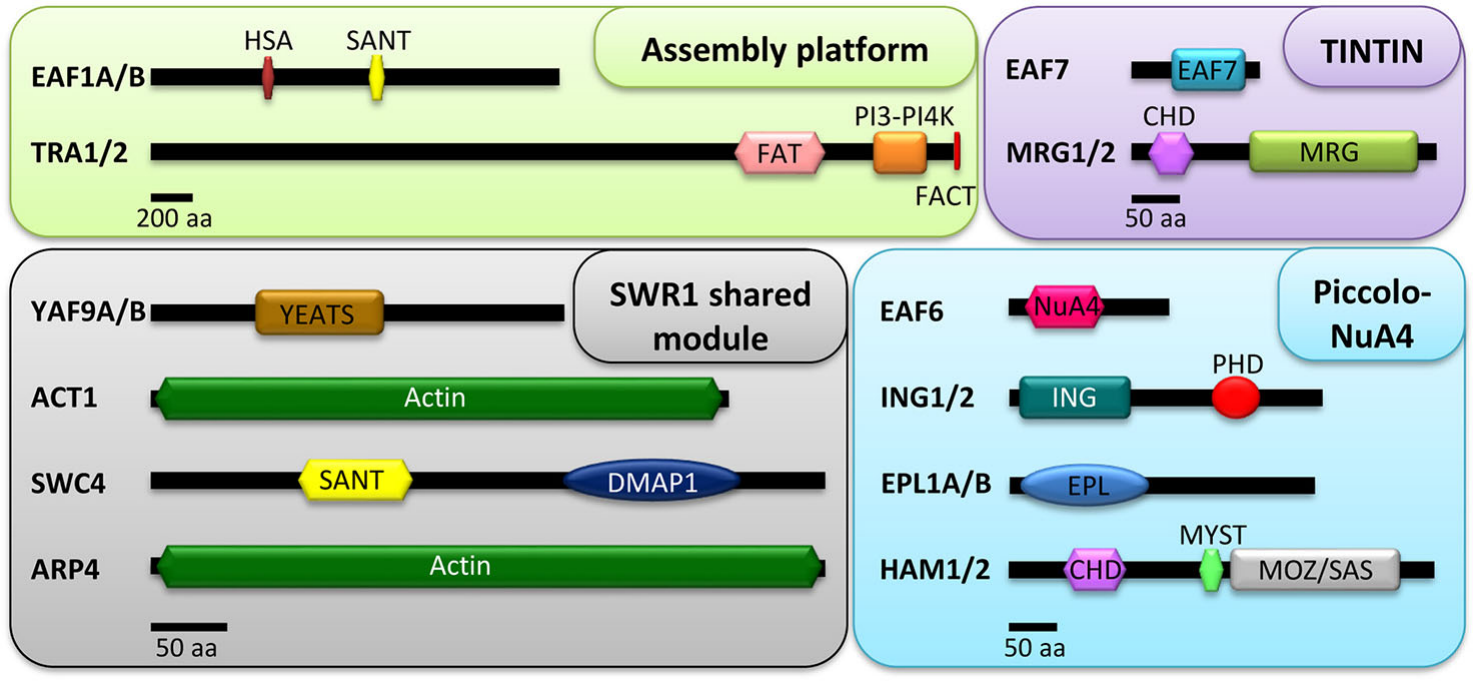
Conserved domains of the putative NuA4 subunits in plants. Proteins are grouped according to the different sub-modules of the complex: assembly platform, Piccolo NuA4, TINTIN, and SWR1 shared module. The modular architecture of the proteins was extracted from multiple alignments with the web servers HMMER v2.1 (Finn et al., 2011) and SMART (Letunic and Bork, 2018). Scale bars are indicated for the proteins of each sub-module. For the assembly platform, 200 aa; for Piccolo NuA4, TINTIN, and the SWR1 shared module, 50 aa.

The existence of a putative NuA4-like complex in plants remains an open question nowadays since this complex has not been purified or characterized yet in any plant species. There are gene homologs for most of the yeast NuA4 subunits in plant genomes, but not for Eaf5 (**Table 1** and **Figure 1**). Many of these genes appear duplicated in the Arabidopsis genome, suggesting that this complex might be also present in plants. However, knowledge concerning the function of the putative plant NuA4 is very limited. Only during the last years the study of Arabidopsis mutants deficient for particular NuA4 subunits has started to reveal functions for these homologs in several biological processes (Latrasse et al., 2008; Umezawa et al., 2013; Bu et al., 2014; Xu et al., 2014; Bieluszewski et al., 2015; Gómez-Zambrano et al., 2018; Peng et al., 2018; Crevillén et al., 2019). Nevertheless, the presence of NuA4 homologs within different multisubunit chromatin remodeling complexes (Latrasse et al., 2008; Bieluszewski et al., 2015; Lin et al., 2017; Tan et al., 2018) may complicate the interpretation of the phenotypic alterations observed in some of these mutants. In any case, a picture is beginning to emerge showing the involvement of various components of this HAT complex in the regulation of a variety of plant biological processes, such as flowering initiation, gametophyte development, cell proliferation, stress, growth, and hormone responses among others (Latrasse et al., 2008; Umezawa et al., 2013; Bu et al., 2014; Xu et al., 2014; Bieluszewski et al., 2015; Gómez-Zambrano et al., 2018; Peng et al., 2018; Crevillén et al., 2019).

As discussed above, Eaf1, together with Tra1, fulfills the role of the assembly platform of the yeast NuA4 (Auger et al., 2008; Wang et al., 2018a). In plants, the Eaf1 subunit is widely conserved and particularly in *A. thaliana* a couple of tandem repeated *EAF1* homologs, *AtEAF1A* (AT3G24880) and *AtEAF1B* (AT3G24870), exist and have already been analyzed (**Table 1** and **Figure 1**). These genes share 98.5% identity in their coding regions and are equally expressed in mature rosette leaves (Bieluszewski et al., 2015). AtEAF1 proteins have also been proposed to function as the scaffold platform of the putative plant NuA4 (Bieluszewski et al., 2015). Besides, Tra1 is also conserved in Arabidopsis and two genes, *AtTRA1A* (AT4G36080) and *AtTRA1B* (AT2G17930), encode homologs of the yeast and the mammalian counterparts (Lu et al., 2009), supporting that the assembly platform for NuA4 is present in plants (**Table 1** and **Figure 1**). The two AtEAF1 proteins contain highly conserved HSA and SANT domains (**Figure 1**), which are also present in the yeast Eaf1 subunit, *H. sapiens* p400 and several plant homologs of PHOTOPERIOD-INDEPENDENT EARLY FLOWERING 1 (PIE1), the proposed catalytic subunit of plant SWR1 (Noh and Amasino, 2003; Bieluszewski et al., 2015). The HSA domain is a common feature of the platform subunits of the chromatin remodeling complexes SWR1, NuA4, and the hybrid complex TIP60-p400 (Szerlong et al., 2008), and is thought to provide the assembly surface for the shared submodule between NuA4 and SWR1 (Auger et al., 2008; Szerlong et al., 2008).

**Figure 2:**
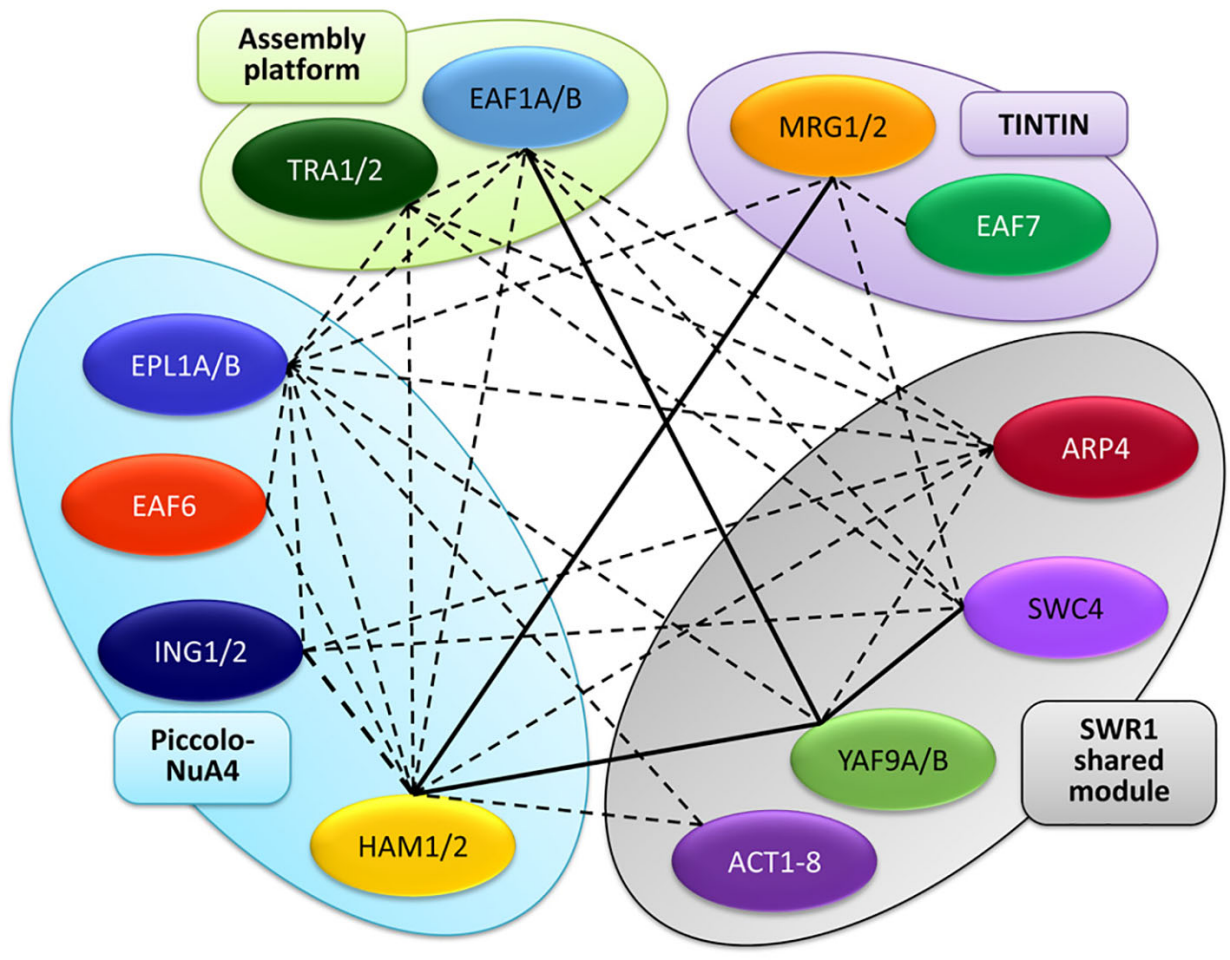
Interaction map among NuA4 subunits in *A. thaliana*. The different homologs are grouped into the different sub-modules of the complex, similarly to **Figure 1**. Continuous lines represent interactions demonstrated by pair-wise protein-protein experiments, whereas dotted lines depict interactions revealed in proteomic experiments.

Besides the conservation of NuA4 subunits, recent works have provided additional evidence for the occurrence of this HAT complex in plants. By using the Arabidopsis homologs of yeast Swc4 and Arp4, AtSWC4 and AtARP4, as baits in affinity purification experiments followed by tandem mass spectrometry (AP-MS/MS), hints for the physical association of these proteins with AtEAF1 were revealed. Interestingly, homologs for the rest of NuA4 subunits, including the other assembly platform protein AtTRA1 (specifically AtTRA1B), were also pulled down in these proteomic assays, suggesting that all these NuA4 components coexist in multimeric complexes in Arabidopsis (**Figure 2**). According to the presence of SWC4 and ARP4 in other chromatin remodeling complexes, subunits of SWR1 and INO80 were also identified (Bieluszewski et al., 2015). Furthermore, co-immunoprecipitation experiments have demonstrated that both YAF9 homologs present in Arabidopsis, AtYAF9A and AtYAF9B, physically interact with AtEAF1B through the HSA domain (**Figure 2**) (Bieluszewski et al., 2015), and that AtYAF9A is also able to interact with AtEAF1A in pulldown assays (Crevillén et al., 2019).

Additional AP-MS experiments have uncovered that both AtEAF1 and AtTRA1 immunoprecipitate as well when other putative subunits of NuA4, including HAM1 and HAM2 (Arabidopsis Esa1 homologs), and the two EPL homologs, AtEPL1A and AtEPL1B, were used as baits (**Figure 2**) (Tan et al., 2018), consistent with a crucial role of both AtEAF1 homologs as assembly platforms for NuA4. Furthermore, these proteomic analyses revealed that not only AtEAF1 but also ten additional conserved subunits of NuA4 were copurified with tagged versions of AtHAMs and AtEPLs in Arabidopsis (**Figure 2**) (Tan et al., 2018). Confirmation for these observations came from Co-IP experiments performed in *N. benthamiana* leaves that demonstrated a physical interaction between AtHAM1 and AtYAF9A proteins *in vivo* (Crevillén et al., 2019). Intriguingly, AtTRA1 homologs were also pulled down in proteomic assays performed using either the SWR1 subunit ARP6 (Sijacic et al., 2019) or the SWR1-interacting protein MBD9 (Methyl-CpG-binding domain 9) (Potok et al., 2019) as baits, further supporting an intricate functional relationship between Arabidopsis SWR1 and NuA4 complexes. Altogether, these observations reinforce our hypothesis that a putative NuA4 exists in plants and may be closely linked with SWR1.

### Emerging Roles of NuA4 in the Control of Plant Biological Processes

**Figure 3:**
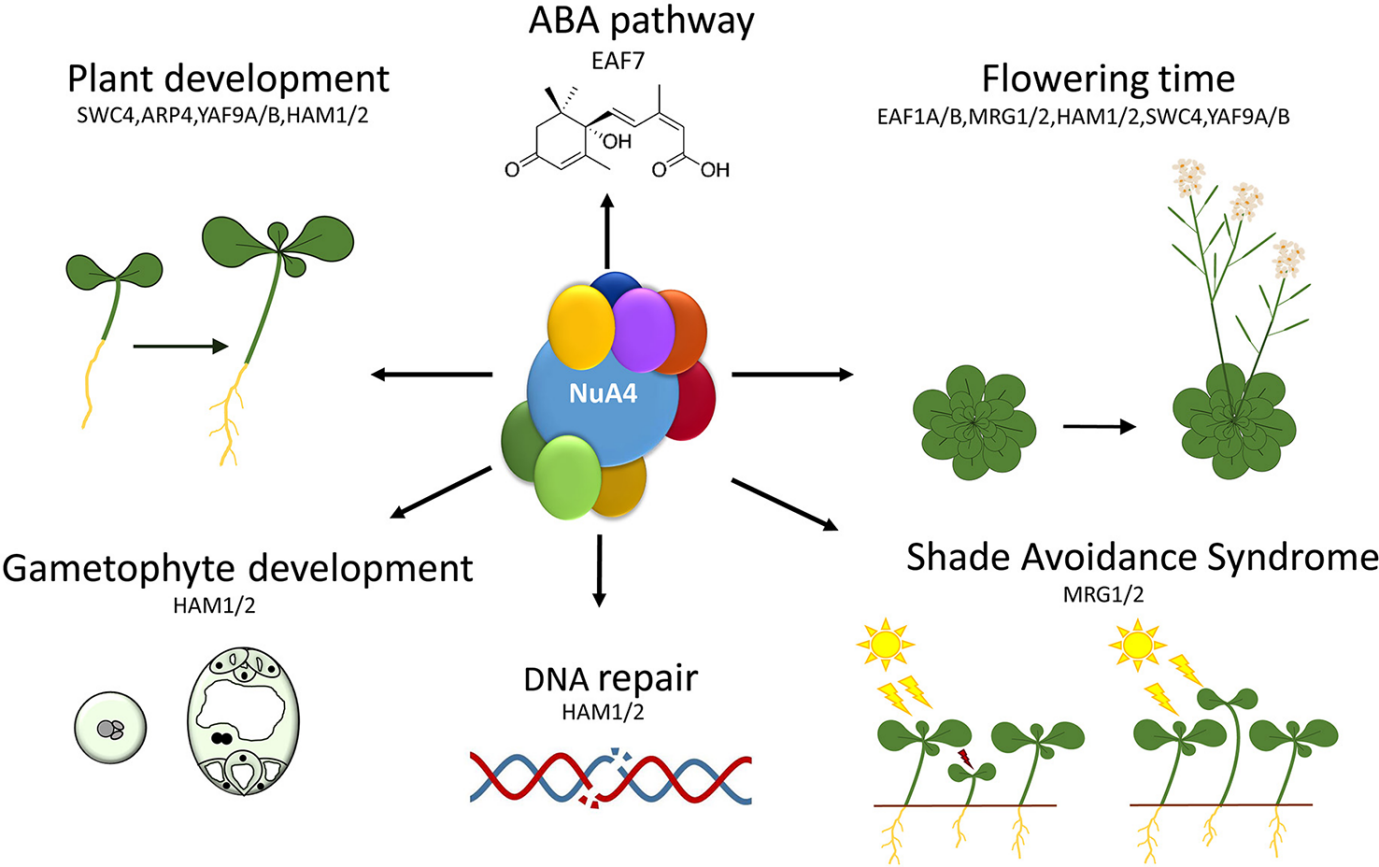
Functions of the putative NuA4 subunits in different plant biological responses. Particular NuA4 subunits are involved in a number of developmental processes such as flowering transition, gametophyte development or hypocotyl growth, as well as in cell proliferation processes and stress responses or in hormone signaling transduction pathways.

The functional characterization of putative Arabidopsis NuA4 components has revealed the involvement of these subunits in the control of a variety of plant biological processes ranging from different aspects of growth and development to stress responses. Interestingly, the emerging picture unveils the implication of a putative plant NuA4 in the regulation of the floral transition, as shown by the abnormal flowering time phenotypes observed in the mutants affected in most of the NuA4 subunits characterized so far (Xiao et al., 2013; Bu et al., 2014; Xu et al., 2014; Bieluszewski et al., 2015; Gómez-Zambrano et al., 2018; Crevillén et al., 2019). This trend strongly argues for a role of NuA4 in the control of plant developmental programs, and particularly, flowering time, a phase transition with important implications in plant adaptation and reproductive success (**Figure 3**).

#### The Piccolo and NuA4 Catalytic Subunits HAM1/2 of Arabidopsis Are Involved in the Control of Developmental Responses

MYST family acetyltransferases have been identified in several plant species. The Arabidopsis genome contains two closely related homologs of the yeast Esa1 protein, HAM1 (AT5G64610) and HAM2 (AT5G09740) (**Table 1** and **Figure 1**) (Latrasse et al., 2008). Their transcripts are constitutively expressed in all tissues, with higher expression levels found in shoot apical meristems, mainly during the floral transition (Earley et al., 2007; Latrasse et al., 2008). In contrast to Arabidopsis, *Solanum lycopersicum* contains only one MYST protein, SlHAM1 (Cigliano et al., 2013). Similar to *AtHAM1* and *AtHAM2*, *SlHAM1* is expressed in all organs, but mainly in flowers and fruits, suggesting that it could accomplish the same developmental role as its Arabidopsis homologs. Phylogenetic analyses have shown that plant HAMs are distributed in two clades, one of which includes both tomato and Arabidopsis proteins while the other comprises two HAM proteins from monocots, including maize and rice. This separation indicates that a single ancestral *HAM* gene gave rise to *HAM* homologs in monocots and dicots, occurring a specific event of duplication at the origin of the expansion of this family in Arabidopsis and maize (Cigliano et al., 2013).

Both HAM1 and HAM2 catalytic subunits have been functionally analyzed (**Figure 3**) and shown to specifically acetylate K5 residues of the histone H4 both *in vitro* and *in vivo* (Earley et al., 2007). The high sequence similarity between HAM1 and HAM2 suggests that a functional redundancy could exist for these proteins (Latrasse et al., 2008; Xiao et al., 2013). Consistent with this, Arabidopsis mutants lacking only one of the HAM proteins do not display noticeable phenotypic alterations when grown under standard conditions. However, *ham1 ham2* double mutants are lethal due to severe defects in the development of male and female gametophytes (**Figure 3**) (Latrasse et al., 2008). Although this embryo lethality hampered the complete functional characterization of *HAM* genes, some pieces of information have been inferred by assessing sesquimutants (Latrasse et al., 2008), double heterozygous mutants (Li et al., 2018), and knockdown and over-expression lines of both *HAM1* and *HAM2* (Xiao et al., 2013). Interestingly, total H4 acetylation levels were reduced in knock-down *ham* lines and increased in HAM1-overexpressors, corroborating that HAM1 functions as HAT *in planta* (Xiao et al., 2013). In addition, *ham1/ham1 ham2/HAM2* and *ham1/HAM1 ham2/ham2* sesquimutant plants display smaller siliques and lower seed number compared to wild-type (wt), as well as unfertilized ovules in some of the analyzed fruits. Furthermore, only 60% of the pollen grains are viable in the anthers of the sesquimutants. These results confirm that both proteins work redundantly to regulate gametophyte development (Latrasse et al., 2008).

Besides its function in gametogenesis, HAM1 and HAM2 also regulate flowering time (**Figure 3**), since *ham* knock-down or *ham1/HAM1 ham2/HAM2* double heterozygous plants in *FRIGIDA* (*FRI*) background (Xiao et al., 2013; Li et al., 2018) displayed an early flowering time phenotype that is accompanied by a reduction in the expression levels of the floral repressors *FLC* and *MADS-BOX AFFECTING FLOWERING GENES 3/4* (*MAF3/4)* (Xiao et al., 2013). These are negative regulators of the floral integrators *SUPPRESSOR OF OVEREXPRESSION OF CO 1* (*SOC1)* and *FT*, the latter being part of the florigen (Andrés and Coupland, 2012). Chromatin immunoprecipitation (ChIP) analyses demonstrated a substantial reduction in H4K5ac and H4ac levels in different regions of *FLC* and *MAF3*/*4* genes in the knock-down transgenic lines, consistent with the low expression levels observed for these flowering genes. In contrast, *HAM* overexpression lines displayed the opposite behavior, showing late flowering, increased levels of *FLC* and *MAF3*/*4*, and higher H4ac levels in these genes. Thus, HAM1 and HAM2 regulate H4 acetylation in the genomic region of these floral repressors, modulating their activation and, consequently, the timing of flowering (Xiao et al., 2013). This is in agreement with previous observations that revealed that flowering time and the expression levels of the floral repressor *FLC* are finely tuned by changes in histone acetylation (revised in He, 2012).

**Figure 4:**
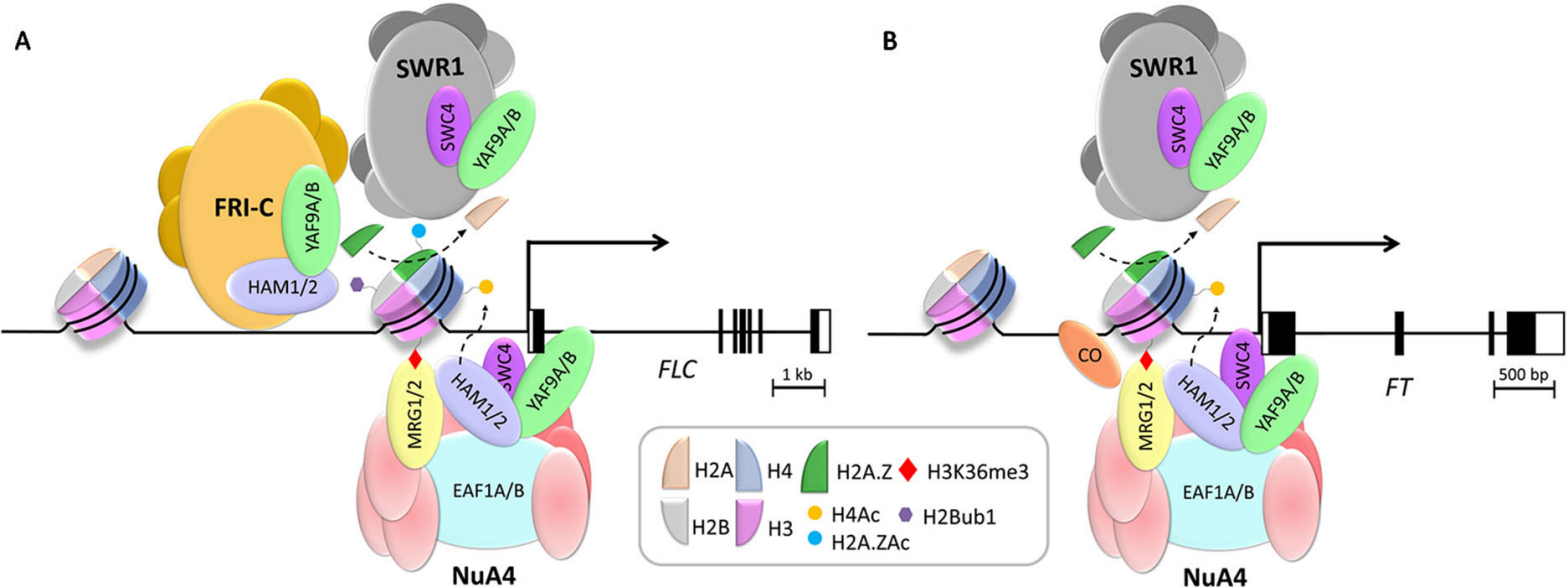
Working models for the NuA4-dependent activation of the flowering time master regulators *FLC* and *FT*. **(A)** H2A.Z deposition mediated by SWR1 and histone H4 and H2A.Z acetylation performed by the putative NuA4 catalytic subunits HAM1/2 in genomic regions surrounding *FLC* TSS are required for *FLC* expression. FRI-C, together with HAM1 and a plethora of chromatin remodelers and transcriptional regulators assist in the recruitment of SWR1 to *FLC* chromatin, fine tuning *FLC* expression by facilitating the incorporation of H2A.Z and histone PTMs. **(B)** HAM1/2 mediate H4K5 acetylation at *FT* chromatin promoting its transcriptional activation. HAM1/2 interact with MRG1/2 proteins as subunits of NuA4. MRGs recognize enriched H3K36me3 regions at *FT* chromatin, and *via* their MRG domain, physically interact with CO to enhance *FT* activation under LD inductive photoperiods. On the other hand, SWR1 mediates the deposition of H2A.Z at the TSS of *FT* chromatin, modulating its expression. See text for details. Scale bars refer to the size of coding regions of the genes from their respective TSS.

On top of being part of NuA4, HAM proteins are associated *in vivo* with components of the PWWPs-EPCRs-ARIDs-TRBs (PEAT) complex (Tan et al., 2018), which mediates histone deacetylation and heterochromatin condensation. Interestingly, a recent study described that HAM1 protein also co-immunoprecipitates with FRI (Li et al., 2018). This flowering regulator defines the FRI complex (FRI-C), which is key to promote *FLC* expression and delay the floral transition. According to these observations, HAM1, recruited together with the FRI-C and a number of chromatin remodeling complexes, is part of the FRI supercomplex (FRI^SC^), which is enriched in the TSS region of *FLC* to mediate its transcriptional activation (**Figure 4A**) (Li et al., 2018). However, it remains to be elucidated if HAM-mediated histone acetylation at the *FLC* locus (Xiao et al., 2013) is functionally related with this FRI^SC^.

In addition to gene transcriptional regulation, histone acetylation is also related to DNA damage repair processes in yeast and mammals (House et al., 2014). In Arabidopsis, the role of HAM1 and HAM2 in DNA damage repair is conserved (**Figure 3**). Single *ham1* and *ham2* mutants display little or no clear phenotypical alterations when grown under normal conditions, but suffer more DNA damage than wt plants when they were exposed to ultraviolet (UV)-B irradiation (Campi et al., 2012). This suggests that plant HAM proteins conserve a functional role in DNA damage repair. In Arabidopsis, HAM1 appears to play a predominant role in this response since *ham1* mutant plants are more affected by UV-B irradiation than those that are defective in *HAM2* (Campi et al., 2012).

The data discussed above support the involvement of HAM proteins in the control of both developmental processes and DNA repair in plants (**Figure 3**). However, the presence of HAM proteins in additional Arabidopsis multiprotein chromatin remodeling complexes hinders the interpretation of the phenotypic alterations observed in *ham* mutants that could be affecting the function of other complexes (Li et al., 2018; Tan et al., 2018), in addition to NuA4. Future studies will contribute to clarify the implication of this HAT complex in the transcriptional control of biological processes and to ascribe specific NuA4-dependent roles for HAM proteins in plants. For that reason, it will be essential to address the implication of additional Arabidopsis Piccolo subunits (EPL1A/B, ING1/2, and EAF6) in the regulation of those processes. In Arabidopsis there are two homologs of yeast Epl1, *AtEPL1A* (AT1G16690) and *AtEPL1B* (AT1G79020) (**Table 1**). Both genes are located in chromosome 1 and are 67% identical with a highly conserved EPL domain (**Figure 1**) (Perry, 2006). EPL proteins are well conserved in plants, and one homolog has been described in maize (*ZmEpl101)* and in other plant species such as soybean, tomato, potato, wheat, and barley (Springer et al., 2002). However, the function of these proteins in plants remains completely unknown.

Piccolo ING homologs are conserved across the plant kingdom and share a similar architecture, with a C-terminal PHD finger module (**Figure 1**). Homologs of ING were found in the moss *Physcomitrella patens*, the monocot *Oryza sativa*, the dicot *Medicago truncatula,* and in the woody plant *Populus trichocarpa* (He et al., 2005; Lee et al., 2009). In Arabidopsis there are two PHD-containing proteins homologs of yeast Yng2 named AtING1 (AT3G24010) and AtING2 (AT1G54390) (**Table 1**) (He et al., 2005; Lee et al., 2009). Both *AtING1* and *AtING2* are ubiquitously expressed, although at low levels. The corresponding proteins are nuclear localized and hold the ability to bind H3K4me2/3 marks, as it has been described in PHD-containing proteins in yeast and mammals, where they can recruit either HAT or HDAC complexes (Lee et al., 2009). In plants, these histone binding modules are involved in the regulation of developmental processes and defense responses among others (Mouriz et al., 2015). However, the function of AtING proteins remains unknown, although a physical interaction of ING2 with Histone Deacetylase Complex 1 (HDC1), an important component of the plant HDAC machinery, has been reported (Perrella et al., 2016). Similarly, the functional roles of AtEAF6, the fourth member of Arabidopsis Piccolo encoded by AT4G14385, have not been defined yet. Further studies are needed to clarify the functions of these putative NuA4 components that accompany HAM proteins in the putative plant Piccolo NuA4 complex.

#### The Assembly Platform Subunit EAF1 Is Also Involved in the Regulation of Plant Development

Further to the roles of the catalytic NuA4 subunit, HAM proteins, recent reports have shed light on the role of AtEAF1 in the control of plant developmental processes (Bieluszewski et al., 2015). Knockout plants for *AtEAF1B* display an acceleration of flowering associated with a reduction in the expression of *FLC* and lower histone acetylation levels near the 5' end of this locus (**Figure 4A**). Besides, these *eaf1b* mutant plants show a reduction in H4K5ac levels in the chromatin of other master regulators of flowering such as *FT*, *CONSTANS* (*CO)* and *SOC1 (* **Figure 4B**) (Bieluszewski et al., 2015). Interestingly, the physical interaction of AtEAF1 with AtYAF9A and AtYAF9B is in agreement with the early flowering phenotype shared by *Ateaf1b*, *Atyaf9a,* and *Atyaf9a Atyaf9b* mutant plants (see below). The acceleration of flowering observed in all these mutants can be attributed to decreased *FLC* expression mainly due to the reduction of its H4 acetylation levels (**Figure 4A**) (Zacharaki et al., 2012; Bieluszewski et al., 2015; Crevillén et al., 2019). Although the location in tandem of both *AtEAF1* homologs in the genome has prevented the isolation of *eaf1a eaf1b* double mutants, the observations regarding *eaf1b* mutants (Bieluszewski et al., 2015) support the involvement of this putative NuA4 subunit in the regulation of flowering time. The alteration of histone acetylation levels displayed by plants deficient in *EAF1* is again consistent with the existence of a functional NuA4 in plants and its participation in developmental regulation.

#### Developmental and Stress-Related Functions Associated to Homologs of Putative Plant TINTIN Complex Subunits

In Arabidopsis there are two homologous proteins for the MRG family, namely AtMRG1 (AT4G37280) and AtMRG2 (AT1G02740) (**Table 1**). Both share nearly 50% identity and 65% similarity in their amino acids sequence. As their human and yeast counterparts, both Arabidopsis proteins conserve the CHD motif that binds H3K4me3 and H3K36me3, and the MRG domain (**Figure 1**) (Bu et al., 2014; Xu et al., 2014; Xie et al., 2015). Arabidopsis *MRG1* and *MRG2* genes are ubiquitously expressed in all tissues but mainly in roots, inflorescences, and the vasculature of cotyledons and true leaves (Bu et al., 2014; Xu et al., 2014). Moreover, the corresponding proteins are nuclear localized, close to euchromatic regions (Xu et al., 2014).

*AtMRG1* and *AtMRG2* are functionally redundant in the control of flowering time in Arabidopsis since plants defective in only one of the *MRG* genes do not display any phenotypic alteration in comparison to wt, while *mrg1 mrg2* double mutant plants show late flowering and low expression levels of the flowering integrator gene *FT* specifically under long-day (LD) conditions, suggesting a link with the photoperiod-dependent flowering pathway (Bu et al., 2014; Xu et al., 2014). In this pathway, the activation of *FT* is critical and promoted by the transcription factor CO in LD. Only under these conditions CO protein is stabilized in the afternoon, allowing the induction of *FT* expression (Song et al., 2015). Interestingly, MRG1/2 proteins physically interact with CO *via* their MRG domain to activate *FT* expression (Bu et al., 2014). In fact, a model of functional interdependence between CO and MRG1/2 has been proposed, in which CO directly binds the *FT* promoter and recruits MRG1/2 proteins to *FT* locus. In addition, the CHDs present in MRG1/2 allow these proteins to bind regions of the *FT* promoter enriched in H3K4me3 and H3K36me3 marks. In this way, MRG1/2 proteins stabilize the binding of CO to the *FT* promoter region, eventually controlling *FT* activation (**Figure 4B**) (Bu et al., 2014). Intriguingly, in addition to regulating *FT*, MRG1 and MRG2 are also needed to fine tune *FLC* expression since *mrg1 mrg2* double mutant plants display reduced expression levels of this floral repressor. The significance of this regulation for the control of flowering time is unclear given that this double mutant is late-flowering, and this delay cannot be explained by low *FLC* expression levels (Xu et al., 2014).

Arabidopsis MRG proteins also interact with HAM1 and HAM2 (Xu et al., 2014). This interaction is conserved through evolution since in yeast, the MYST HAT Esa1 associates with Eaf3 to specifically target gene promoter regions for transcriptional activation (Eisen et al., 2001). *mrg1 mrg2* double mutants show a reduction in acetylation levels of histone H3 and histone H4K5 at their target genes, and particularly in *FT* (Xu et al., 2014). Therefore, according to the current working model, MRG1 and MRG2 preferably bind H3K36me3 at the promoter region of their target genes and recruit HATs HAM1/2 to increase histone acetylation levels, making these loci more accessible for transcriptional activation (**Figure 4B**) (Xu et al., 2014).

MRG1 and MRG2 also function as positive regulators of shade-induced hypocotyl elongation responses (**Figure 3**) (Peng et al., 2018). Plants grown in high-density conditions adapt their development to ensure its accessibility to sunlight. This is known as shade avoidance syndrome (SAS), where the most characteristic phenotypic response is hypocotyl elongation (Ballaré and Pierik, 2017). Plants defective in both *MRG1* and *MRG2* are affected in SAS response since they display a shorter hypocotyl length compared to wt plants when they are grown under shade, while there are no differences under standard white light or dark conditions (Peng et al., 2018). PHYTOCHROME-INTERACTING FACTOR 7 (PIF7) also plays a crucial role in this response, since *pif7* mutants display shorter hypocotyls only when they are grown under shade conditions (Li et al., 2012). Interestingly, MRG2 and PIF7 proteins physically interact, and in response to shade PIF7 recruits MRG1/2 to the promoter of the target loci to regulate the expression of shade-responsive genes (Peng et al., 2018). In turn, MRG1/2 proteins bind H3K4me3 and H3K36me3 marks and recruit HAT complexes to promote histone acetylation, inducing the expression of genes mediating SAS (Peng et al., 2018).

Arabidopsis MRG proteins have also been implicated in the flowering response to ambient temperature (Pajoro et al., 2017), although it is currently unclear whether this function of MRGs is linked to NuA4 activity. In Arabidopsis, genome-wide approaches have shown that genes differentially spliced in response to fluctuating ambient temperature are enriched in H3K36me3 (Pajoro et al., 2017). Interestingly, Arabidopsis *mrg1 mrg2* mutant plants are less sensitive to the high ambient temperature acceleration of flowering observed in wt plants, suggesting that MRG1/2 could mediate the H3K36me3-dependent regulation of alternative splicing that occurs under warm temperature (Pajoro et al., 2017). Based on these observations, it is tempting to speculate that a link might exist between the activity of NuA4 and alternative splicing in response to environmental cues. However, it is not possible to rule out that this role of MRG proteins is NuA4-independent, and further research will be necessary to explore the implication of this HAT complex in mRNA maturation in plants.

MRG proteins are conserved across the plant kingdom, and their function has been addressed not only in Arabidopsis but also in other plant species. The rice genome contains two *MRG* genes, *OsMRG701* and *OsMRG702* (**Table 1**), the latter being involved in the floral transition since knockdown mutants for this gene display a late flowering phenotype under both LD and short-day (SD) conditions (Jin et al., 2015). Interestingly, this mutant shows similar developmental alterations to those observed in brassinosteroid (BR)-deficient plants. These defects are related to the ability of MRG702 to bind chromatin and regulate the expression of BR biosynthesis genes. Like other MRG family proteins, MRG702 also directly binds the chromatin of target genes in an H3K36me3-dependent manner (Jin et al., 2015).

On the other hand, AT1G26470 encodes AtEAF7 in Arabidopsis, the homologous protein of yeast Eaf7 and human MRGBP (**Table 1** and **Figure 1**) (Ito et al., 2018). AtEAF7 is also known as SNS1 (SnRK2-SUBSTRATE 1) since it is a target of SnRK2 (Umezawa et al., 2013), a protein kinase involved in abscisic acid (ABA) signaling pathway (Hirayama and Umezawa, 2010). In response to ABA, SnRK2 is activated and phosphorylates a variety of protein substrates, including AtEAF7/SNS1 (Umezawa et al., 2013). Plants deficient in *AtEAF7/SNS1* display a conspicuous inhibition of post-germination growth when they are grown in the presence of ABA, while they grow normally in the absence of this phytohormone (Umezawa et al., 2013). In comparison to wt, ABA-responsive genes appeared upregulated in *Ateaf7/sns1* seedlings treated with ABA. Thus, AtEAF7/SNS1 has been proposed to act as a negative regulator of ABA signaling pathway in Arabidopsis at the post-germination stage (**Figure 3**) (Umezawa et al., 2013).

Although evidence for the physical interaction between AtEAF7 and MRG1/2 is still lacking, the dimers between AtEAF7 and MRG1/2 might work in plants as a functional homolog of the TINTIN complex with both NuA4-dependent and -independent functions. Given the role of AtEAF7 in the ABA signaling pathway (Umezawa et al., 2013), it will be interesting to investigate the possible implication of MRG1/2 in abiotic stress responses mediated by this hormone. In addition, emerging evidence has unveiled the participation of alternative splicing mechanisms in ABA-mediated responses (Laloum et al., 2018). Besides, SnRK2 kinases have been shown to regulate the phosphorylation status of several plant splicing factors (Laloum et al., 2018), suggesting a link between these two processes that might implicate Arabidopsis homologs of the TINTIN subunits, an issue that will need to be thoroughly explored in the future.

#### Accessory Subunits of NuA4 Shared With SWR1 Also Regulate Developmental Programs

Arabidopsis SWR1 is involved in the control of plant stress responses and developmental processes, particularly in the regulation of flowering time. Mutations affecting different SWR1 subunits cause an acceleration of flowering due to reduced *FLC* expression (Jarillo and Piñeiro, 2015). Consistently, loss of function mutants in the genes encoding different isoforms of H2A.Z in Arabidopsis display similar phenotypes (Coleman-Derr and Zilberman, 2012), indicating that SWR1 is required to control flowering time mainly through the deposition of H2A.Z histone variant in regulatory regions of *FLC* (Martín-Trillo et al., 2006; Deal et al., 2007; Jarillo and Piñeiro, 2015). Arabidopsis homologs of the four shared subunits between SWR1 and NuA4 complexes (YAF9, SWC4, ARP4, and ACT1) have been characterized, revealing that their loss of function confers in most of them pleiotropic phenotypic alterations similar to other *swr1* mutants.

Two genes encoding YEATS domain-containing homologs to the yeast Yaf9 are present in the Arabidopsis genome, *AtYAF9A* (AT5G45600) and *AtYAF9B* (AT5G18000) (**Table 1** and **Figure 1**) (Zacharaki et al., 2012; Bieluszewski et al., 2015; Crevillén et al., 2019). The YEATS domain of Yaf9 has been defined as a selective reader of H3K27ac in yeast, and the recognition of this histone modification by Yaf9 leads to the exchange of the H2A histone variant for H2A.Z (Klein et al., 2018). In Arabidopsis, both YAF9A/B proteins can recognize unmodified histone H3 and also H3K9ac and H3K27ac, suggesting that the ability of the YEATS domain to bind acetylated H3 is conserved in plants and could participate in the recruitment of YAF9 to chromatin (Crevillén et al., 2019). *YAF9A* is highly expressed in different organs of the plant, while high expression levels of *YAF9B* were only detected in young flowers and roots (Zacharaki et al., 2012; Crevillén et al., 2019). The proteins encoded by both genes are located in the nucleus, consistent with YAF9 proteins being present in chromatin remodeling complexes (Crevillén et al., 2019). Plants deficient in *YAF9A* display a slight but significant acceleration of flowering in both LD and SD. However, plants defective in *YAF9B* behave like wt (Crevillén et al., 2019). Interestingly, *yaf9a yaf9b* double mutant plants show pleiotropic developmental phenotypic alterations in comparison to wt plants, such as accelerated senescence and chlorotic leaves with reduced chlorophyll content, conspicuous early flowering under both LD and SD conditions, and reduced organ and plant size due to lower endoreduplication levels (Bieluszewski et al., 2015; Crevillén et al., 2019). Results derived from transcriptomic analyses with *yaf9a yaf9b* plants are in agreement with their pleiotropic phenotypic alterations. These mutants show more than 2000 differentially expressed genes, including some related to cell size and growth regulation, systemic acquired response, and also to flowering time regulation (Crevillén et al., 2019). Altogether, these data indicate that the Arabidopsis *YAF9A* and *YAF9B* genes have partially redundant roles in the regulation of developmental processes, including the initiation of reproductive growth.

According to their early flowering, both *yaf9a* and *yaf9a yaf9b* plants have reduced *FLC* transcript levels, while in *yaf9b* no alterations are observed in *FLC* expression (Zacharaki et al., 2012; Bieluszewski et al., 2015; Crevillén et al., 2019). Interestingly, crosses of *flc* plants with *yaf9a* or *yaf9a yaf9b* reveal an additive effect between the corresponding genes, suggesting that *YAF9* genes regulate this developmental transition through both *FLC*-dependent and -independent mechanisms (Crevillén et al., 2019). The downstream floral integrators *FT* and *SOC1* are upregulated in *yaf9a* and *yaf9a yaf9b* mutants, consistent with the early flowering observed in these plants (Zacharaki et al., 2012; Bieluszewski et al., 2015; Crevillén et al., 2019).

In Arabidopsis, SWR1 is necessary for the activation of *FLC via* the exchange of H2A by the histone variant H2A.Z in the chromatin of this locus (Martín-Trillo et al., 2006; Deal et al., 2007). However, no change in H2A.Z levels were found between wt and *yaf9a yaf9b* double mutant plants at *FLC* chromatin, suggesting that YAF9 proteins are not required for H2A.Z deposition at this locus, and that YAF9 proteins also regulate flowering in an SWR1-independent manner. Supporting this conclusion, the combination of *swr1* mutants with *yaf9a* and *yaf9a yaf9b* revealed an additive effect on flowering time (Crevillén et al., 2019). *yaf9a yaf9b* double mutants also show a distinct genetic interaction with *FRI* in comparison with other subunits of SWR1. As discussed above, the FRI-C delays flowering by promoting *FLC* expression (Choi et al., 2011; Crevillén and Dean, 2011; Li et al., 2018). When combined with *FRI* alleles, *yaf9a yaf9b* mutations partially suppress this late flowering and the high expression levels of *FLC*. This is in contrast with the flowering time phenotype observed in lines carrying an active *FRI* allele introgressed into other *swr1* mutants, where the suppression of the *FRI* late-flowering phenotype is complete (Choi et al., 2005). These observations corroborate that *FRI* requires an active SWR1 to regulate *FLC* expression, and further support the notion that YAF9 also regulates *FLC* expression *via* SWR1-independent mechanisms (**Figure 4A**) (Crevillén et al., 2019).

ChIP experiments demonstrated that *FLC* is a direct target of YAF9, and that the effect of this protein on *FLC* expression is mediated by changes in chromatin organization since the acetylation levels of both histone H4 and the histone variant H2A.Z were decreased in this locus in *yaf9a* and *yaf9a yaf9b* mutants (**Figure 4A**) (Zacharaki et al., 2012; Bieluszewski et al., 2015; Crevillén et al., 2019). These results reveal for the first time in plants a possible link between H2A.Z acetylation and gene expression that will have to be analyzed in detail in future works. *FT* is also a direct target of YAF9 (**Figure 4B**) (Crevillén et al., 2019), although its expression was upregulated in the *yaf9a yaf9b* double mutant. Despite this increased expression, levels of histone H4ac are moderately reduced at the *FT* locus in these mutant plants (Bieluszewski et al., 2015). In contrast to the *FLC* gene, no changes in H2A.Zac levels were observed in *FT* chromatin in *YAF9* defective plants (Crevillén et al., 2019), revealing that the role of YAF9 in H2A.Z modification could be locus specific and highlighting the complexity of chromatin-mediated regulation of gene expression in plants.

YAF9A also participates in the control of flowering time through the photoperiod-dependent pathway. This NuA4 subunit interacts with the circadian clock component CIRCADIAN CLOCK ASSOCIATED 1 (CCA1) which recruits MUT9P-LIKE-KINASE 4 (MLK4) to the *GIGANTEA* (*GI*) promoter. This protein complex that contains YAF9 and MLK4 acts to induce *GI* expression through phosphorylation of histone H2A at serine 95, H2A.Z deposition, and histone H4 acetylation in the chromatin of this flowering locus (Su et al., 2017). These observations illustrate how the coordinated action of different histone marks and chromatin remodeling complexes establishes patterns of gene expression required for an appropriate regulation of developmental processes such as flowering time.

Another NuA4 subunit shared with SWR1 is SWC4. Arabidopsis SWC4, encoded by AT2G47210, was recently found associated with SWR1 in plants (Gómez-Zambrano et al., 2018) (**Table 1**). Like its yeast and mammalian counterparts, AtSWC4 contains a SANT/Myb_DMAP1 domain in N-terminal position and a DMAP1 domain in the C-terminus (**Figure 1**) (Gómez-Zambrano et al., 2018). The first one is involved in the interaction with DNA, histones, and other proteins, while the second one mediates protein-protein interactions (Zhou et al., 2010). As in yeast, AtSWC4 physically interacts with both AtYAF9A and AtYAF9B in the nucleus (Bieluszewski et al., 2015; Gómez-Zambrano et al., 2018), suggesting the conservation of this common submodule in plants. *SWC4* is widely expressed but shows higher transcript levels in proliferating tissues including roots, flowers, and floral buds, and participates in the regulation of different developmental processes. This NuA4 subunit seems to be essential for Arabidopsis embryo development given that *swc4* knockout mutant plants are embryo-lethal. Furthermore, SWC4 also takes part in the regulation of post-embryonic processes since plants with reduced levels of *SWC4* expression (*swc4i*) grown under both LD and SD conditions display pleiotropic phenotypic alterations in both vegetative and reproductive development such as curly leaves, symptoms of accelerated leaf senescence, and reduced plant and organ size due to a defective balance between cell proliferation and expansion. Additionally, consistent with the early flowering phenotype of *yaf9* mutants, *SWC4* knock-down lines displayed a slight acceleration of flowering concomitantly with *FT* upregulation under LD conditions (Gómez-Zambrano et al., 2018). Many of these morphological and developmental alterations resemble those observed in several Arabidopsis mutants defective in SWR1 function (Choi et al., 2005; Deal et al., 2005; Martín-Trillo et al., 2006; Choi et al., 2007; Deal et al., 2007; Lázaro et al., 2008; Jarillo and Piñeiro, 2015; Crevillén et al., 2019).

Transcriptomic analyses have revealed the misregulation of a wide range of genes in the *SWC4* knockdown plants, which is consistent with the pleiotropic phenotypic alterations observed in these lines. Among the differentially expressed genes in *swc4i* lines, upregulated transcripts were three times more frequent than those downregulated, suggesting a role for SWC4 in gene silencing (Gómez-Zambrano et al., 2018). Differentially expressed genes include some loci involved in primary and secondary metabolism, response to stimulus and stress, post-embryonic development, cell-cycle control, cell differentiation, and growth (Gómez-Zambrano et al., 2018). Interestingly, this transcriptomic analysis revealed a wider overlap with that of the *yaf9a yaf9b* plants than with those of mutants affected in other SWR1 subunits like *pie1*, *arp6,* or *swc6* (Crevillén et al., 2019), suggesting that AtYAF9 and AtSWC4 share a number of functions, and both may have additional roles to those performed by SWR1. In agreement with this idea and similarly to *yaf9a yaf9b* mutant plants (Crevillén et al., 2019), *SWC4* knock-down plants show lower endoreduplication levels (Gómez-Zambrano et al., 2018). This is consistent with YAF9 proteins and SWC4 being part of the same functional submodule. Nevertheless, a significant overlap was still noticeable between the RNA-seq data of *swc4i* lines and transcriptomic profiles of other *swr1* mutants (Gómez-Zambrano et al., 2018; Crevillén et al., 2019). Moreover, ChIP-seq analyses performed in *swc4i* plants identified more than 5000 genes with reduced levels of H2A.Z. Interestingly, these loci significantly overlap with the upregulated genes in *SWC4* deficient plants, consistent with the association of AtSWC4 with SWR1. Based on these results, SWC4 has been proposed to be necessary for the recruitment of SWR1 and H2A.Z deposition to specific loci through the recognition of AT-rich DNA elements that are over-represented in the TSS of target genes. Consistent with previous observations showing that H2A.Z deposition is essential for proper transcriptional regulation of *FT* (Kumar et al., 2012), this master gene of flowering is a direct target of AtSWC4, and is one of the most highly upregulated genes in *swc4i* plants (Gómez-Zambrano et al., 2018), underscoring the relevance of SWR1 activity for modulating floral initiation in Arabidopsis (**Figure 4B**).

Finally, another shared member of SWR1 and NuA4 complexes that has been characterized in Arabidopsis is ARP4 (**Table 1** and **Figure 1**). In Arabidopsis, there are eight classes of ARPs (ARP2-9), and two of them (ARP7 and 8) are plant-specific, while for the rest there are homologs in other eukaryotes. ARP proteins are well conserved in diverse angiosperms and homologs for all ARP classes have been found in the monocot rice (**Table 1**) (Kandasamy et al., 2003). All the Arabidopsis ARP classes are represented by a single gene except for *ARP4*, which has two closely related genes dubbed as *AtARP4* (AT1G18450) and *AtARP4A* (AT1G73910). While *AtARP4A* appears to be poorly expressed, *AtARP4* mRNA is ubiquitously present in all organs and tissues analyzed (Kandasamy et al., 2003). *AtARP4* knockdown plants display morphological alterations like reduction in plant size, smaller and fewer leaves, and atrophied siliques with few seeds and fertility. These plants are also affected in many phases of reproductive development since they display conspicuous alterations in flower development and an early flowering time phenotype specifically under LD, suggesting a possible link with the photoperiodic flowering pathway. This global impact of *AtARP4* deficiency in plant growth and architecture indicates that this gene is involved in the control of several developmental programs (Kandasamy et al., 2005).

ARP4 homologs from Arabidopsis and *Brassica* and tobacco species are nuclear proteins (Kandasamy et al., 2005) and co-purify with multiple putative subunits of INO80, NuA4, SWR1, and SWI/SNF complexes, confirming that ARP4 is part of several nuclear chromatin remodeling complexes in plants. Again, the presence of this protein in multiple complexes may explain the broad range of phenotypic alterations displayed by plants deficient in *ARP4* function. Finally, up to eight isoforms of Act are encoded in the Arabidopsis genome (**Table 1**) (Meagher et al., 2005), but any experimental evidence shedding light on which ones might be involved in the plant NuA4 is still missing.

### The Putative NuA4-SWR1 Complexes Merge in Plants, an Evolutionary Perspective

The existence of a functional interplay between the yeast SWR1 and NuA4 chromatin remodeling complexes has been suggested, based on several observations (Billon and Côté, 2013). First, four subunits are shared by both complexes (Altaf et al., 2010). Second, the NuA4-mediated acetylation of H2A and H4 facilitates the replacement of H2A-H2B with H2A.Z-H2B dimers by SWR1 (Altaf et al., 2010). Third, yeast NuA4 is responsible for the acetylation of H2A.Z histone variant after its incorporation into chromatin by SWR1 (Millar et al., 2006). Fourth, NuA4-mediated histone acetylation and H2A.Z deposition are intimately associated in a number of chromatin remodeling processes such as the establishment of heterochromatin boundaries or the activation of expression in subtelomeric regions (Zhou et al., 2010). Finally, in metazoans, homologs of SWR1 and NuA4 form the hybrid TIP60 complex, which is able to acetylate H2A and H4 histones and, at the same time, exchange H2A with H2A.Z (Cai et al., 2003).

Further to the functional link between SWR1 and NuA4 in yeast and animals, recent reports have demonstrated how these complexes can merge and separate during the transition from unicellular yeast to multicellular hypha in *Candida albicans* (Wang et al., 2018b). During the yeast state of this human pathogen, the catalytic subunit of NuA4 mediates the specific acetylation of the K173 residue in Eaf1. This modification of the NuA4 assembly platform subunit allows the interaction with Yaf9, a shared subunit of NuA4 and SWR1, facilitating the merge of both complexes. In contrast, during hyphal elongation, the acetylation levels of Eaf1 decrease through the action of the histone deacetylase Hda1, which is recruited to chromatin in response to nutritional signals that sustain hyphal elongation. In this state, NuA4 and SWR1 complexes are separated, showing the relevance of the dynamic merge and separation of these complexes in developmental transitions that take place depending on the nutritional status of the fungus (Wang et al., 2018b).

Based on these observations, a plausible scenario is that the merge of SWR1 and NuA4 complexes occurs also in plants. However, compelling experimental evidence supports the notion that plants, from mosses to angiosperms, are most likely to have canonical SWR1 and NuA4 complexes similar to those found in yeast. Most AP–MS approaches using subunits of SWR1 as baits reveal enrichments in SWR1 components and shared subunits with NuA4 among the co-immunoprecipitated proteins. In addition to these proteins, only homologs for the yeast Tra1, present in both NuA4 and SAGA HAT complexes, were recovered in these immunoprecipitation experiments when the SWR1 specific subunit ARP6 was used as bait, but not the NuA4 scaffold (EAF1) nor the catalytic subunit (HAM) (Potok et al., 2019; Sijacic et al., 2019). Based on these results, it is unlikely that these complexes may represent a merge of the Arabidopsis SWR1 and NuA4 complexes, similar to the mammalian TIP60 complex (Cai et al., 2003). In support of this conclusion, the K residue found to be acetylated in *C. albicans* Eaf1 during the transition from yeast to hyphae is not conserved in Arabidopsis, suggesting that this mechanism of separation and merge could be a specific adaptation of polymorphic fungi (Wang et al., 2018b). However, at present we cannot completely rule out the possibility that specific subsets of SWR1, NuA4 or even TIP60-like complexes could establish various combinations of the different homolog subunits that are encoded in the Arabidopsis genome for SWR1 and NuA4 complexes (Gómez-Zambrano et al., 2018) depending on the cell type, the developmental stage or the environmental and growth conditions that plants are exposed to. To precisely address the possible occurrence of distinct SWR1 and TIP60-like complexes, further complex purification approaches using for example Arabidopsis PIE1, the putative catalytic subunit of SWR1, as bait could contribute to elucidate the possible merge of NuA4 and SWR1 in plants.

## Concluding Remarks

Over the last few years, several reports have started to enlighten the possible existence of a functional NuA4 in plants. At least 12 out of the 13 subunits of this complex are conserved in plants, and their functional characterization is providing evidence for the participation of this complex in different developmental processes and environmental responses. However, as for other plant chromatin remodeling complexes, one of the main bottlenecks in the characterization of the putative plant NuA4 is its purification and further crystallization. At the moment, the only information available to support the presence of NuA4 in plants is based on proteomic and individual protein-protein interaction analyses performed in Arabidopsis. While the data gathered until now tend to suggest that most of the components of the complex associate to each other *in vivo*, further complex purification approaches will be needed to clarify the exact biochemical composition of plant NuA4.

Remarkably, most of the NuA4 subunits are duplicated in plants. The functional characterization performed for some of these subunits indicate that different levels of redundancy are found in these couples of paralogs. The combinatorial potential of these homologs either in the full NuA4 complex or in the subcomplexes like Piccolo or TINTIN, is considerable. A plethora of distinct NuA4 chromatin remodelers with specific acetylation properties and functions could be produced in response to environmental factors or developmental cues, increasing plant plasticity that may result in a ﬁtness beneﬁt. Future research is expected to shed light on the possible interplay of the putative plant NuA4 and SWR1 complexes, although no clear evidence for their merge in a TIP60-like complex has been reported so far in Arabidopsis. In fact, current experimental data supports that plants most likely have independent NuA4 and SWR1 complexes, as it happens in yeast. Nevertheless, it is still possible that particular growing conditions, nutritional status, differentiation states or developmental signals may promote the combination of subunits from both complexes in plants, as it has been described in some fungi.

Although knowledge on the function of plant NuA4 is still in its infancy, the study of mutants affected in different NuA4 subunits characterized so far has revealed a number of phenotypic alterations at both vegetative and reproductive stages, suggesting an involvement of NuA4 in the control of central plant developmental programs through acetylation-mediated regulation of gene expression. However, the presence of some of the NuA4 homologs within different multisubunit chromatin remodeling complexes hampers the interpretation of the phenotypic alterations observed in these mutants, or in the combinations between them, complicating at the moment the adscription of functions to particular complexes. Further work is necessary to characterize additional plant NuA4 subunits in order to discriminate the functions that rely on the HAT activity of this complex from those that depend partially or totally on other chromatin remodeling complexes that share components with NuA4. Future comparative genomic and epigenomic analyses concerning mutants affected in specific and non-specific plant NuA4 subunits will allow us to conclude the mechanisms through which NuA4 works in gene expression regulation and the identification of its direct targets, increasing our understanding on how plant NuA4 functions in different developmental programs and environmental responses, and how this complex interacts with other chromatin remodeling activities.

## Author Contributions

JJ and MP suggested and designed the article. LE-C, LB-M, JJ, and MP wrote the paper and designed the figures. JB-G, VJ-S, AL, and RP made valuable suggestions for the manuscript. All authors checked and confirmed the final version of the manuscript.

## Conflict of Interest

The authors declare that the research was conducted in the absence of any commercial or financial relationships that could be construed as a potential conflict of interest.

## References

[B1] Aguissa-Touré A. H. Wong R. P. Li G. (2011). The ING family tumor suppressors: from structure to function. Cell Mol. Life Sci. 68 (1), 45–54. 10.1007/s00018-010-0509-1 20803232 PMC11114739

[B2] Allard S. Utley R. T. Savard J. Clarke A. Grant P. Brandl C. J. (1999). NuA4, an essential transcription adaptor/histone H4 acetyltransferase complex containing Esa1p and the ATM-related cofactor Tra1p. EMBO J. 18 (18), 5108–5119. 10.1093/emboj/18.18.5108 10487762 PMC1171581

[B3] Altaf M. Auger A. Monnet-Saksouk J. Brodeur J. Piquet S. Cramet M. (2010). NuA4-dependent acetylation of nucleosomal histones H4 and H2A directly stimulates incorporation of H2A.Z by the SWR1 complex. J. Biol. Chem. 285 (21), 15966–15977. 10.1074/jbc.M110.117069 20332092 PMC2871465

[B4] Andrés F. Coupland G. (2012). The genetic basis of flowering responses to seasonal cues. Nat. Rev. Genet. 13 (9), 627–639. 10.1038/nrg3291 22898651

[B5] Auger A. Galarneau L. Altaf M. Nourani A. Doyon Y. Utley R. T. (2008). Eaf1 is the platform for NuA4 molecular assembly that evolutionarily links chromatin acetylation to ATP-dependent exchange of histone H2A variants. Mol. Cell Biol. 28 (7), 2257–2270. 10.1128/MCB.01755-07 18212047 PMC2268442

[B6] Avvakumov N. Lalonde M. E. Saksouk N. Paquet E. Glass K. C. Landry A. J. (2012). Conserved molecular interactions within the HBO1 acetyltransferase complexes regulate cell proliferation. Mol. Cell Biol. 32 (3), 689–703. 10.1128/MCB.06455-11 22144582 PMC3266594

[B7] Ballaré C. L. Pierik R. (2017). The shade-avoidance syndrome: multiple signals and ecological consequences. Plant Cell Environ. 40 (11), 2530–2543. 10.1111/pce.12914 28102548

[B8] Bannister A. J. Kouzarides T. (2011). Regulation of chromatin by histone modifications. Cell Res. 21 (3), 381–395. 10.1038/cr.2011.22 21321607 PMC3193420

[B9] Barnes C. E. English D. M. Cowley S. M. (2019). Acetylation & Co: an expanding repertoire of histone acylations regulates chromatin and transcription. Essays Biochem. 63 (1), 97–107. 10.1042/EBC20180061 30940741 PMC6484784

[B10] Berg M. D. Genereaux J. Karagiannis J. Brandl C. J. (2018). The pseudokinase domain of *Saccharomyces cerevisiae* Tra1 is required for nuclear localization and incorporation into the SAGA and NuA4 complexes. G3 (Bethesda) 8 (6), 1943–1957. 10.1534/g3.118.200288 29626083 PMC5982823

[B11] Berr A. Shafiq S. Shen W. H. (2011). Histone modifications in transcriptional activation during plant development. Biochim. Biophys. Acta 1809 (10), 567–576. 10.1016/j.bbagrm.2011.07.001 21777708

[B12] Bhat W. Ahmad S. Côté J. (2015). TINTIN, at the interface of chromatin, transcription elongation, and mRNA processing. RNA Biol. 12 (5), 486–489. 10.1080/15476286.2015.1026032 25775193 PMC4615837

[B13] Bhaumik S. R. Smith E. Shilatifard A. (2007). Covalent modifications of histones during development and disease pathogenesis. Nat. Struct. Mol. Biol. 14 (11), 1008–1016. 10.1038/nsmb1337 17984963

[B14] Bieluszewski T. Galganski L. Sura W. Bieluszewska A. Abram M. Ludwikow A. (2015). AtEAF1 is a potential platform protein for Arabidopsis NuA4 acetyltransferase complex. BMC Plant Biol. 15, 75. 10.1186/s12870-015-0461-1 25849764 PMC4358907

[B15] Billon P. Côté J. (2013). Precise deposition of histone H2A.Z in chromatin for genome expression and maintenance. Biochim. Biophys. Acta 1819 (3-4), 290–302. 10.1016/j.bbagrm.2011.10.004 24459731

[B16] Bird A. W. Yu D. Y. Pray-Grant M. G. Qiu Q. Harmon K. E. Megee P. C. (2002). Acetylation of histone H4 by Esa1 is required for DNA double-strand break repair. Nature 419 (6905), 411–415. 10.1038/nature01035 12353039

[B17] Bittner C. B. Zeisig D. T. Zeisig B. B. Slany R. K. (2004). Direct physical and functional interaction of the NuA4 complex components Yaf9p and Swc4p. Eukaryot. Cell 3 (4), 976–983. 10.1128/EC.3.4.976-983.2004 15302830 PMC500879

[B18] Black J. C. Van Rechem C. Whetstine J. R. (2012). Histone lysine methylation dynamics: establishment, regulation, and biological impact. Mol. Cell 48 (4), 491–507. 10.1016/j.molcel.2012.11.006 23200123 PMC3861058

[B19] Boudreault A. A. Cronier D. Selleck W. Lacoste N. Utley R. T. Allard S. (2003). Yeast enhancer of polycomb defines global Esa1-dependent acetylation of chromatin. Genes Dev. 17 (11), 1415–1428. 10.1101/gad.1056603 12782659 PMC196073

[B20] Bruzzone M. J. Grünberg S. Kubik S. Zentner G. E. Shore D. (2018). Distinct patterns of histone acetyltransferase and Mediator deployment at yeast protein-coding genes. Genes Dev. 32 (17-18), 1252–1265. 10.1101/gad.312173.118 30108132 PMC6120713

[B21] Bu Z. Yu Y. Li Z. Liu Y. Jiang W. Huang Y. (2014). Regulation of Arabidopsis flowering by the histone mark readers MRG1/2 *via* interaction with CONSTANS to modulate *FT* expression. PloS Genet. 10 (9), e1004617. 10.1371/journal.pgen.1004617 25211338 PMC4161306

[B22] Cai Y. Jin J. Tomomori-Sato C. Sato S. Sorokina I. Parmely T. J. (2003). Identification of new subunits of the multiprotein mammalian TRRAP/TIP60-containing histone acetyltransferase complex. J. Biol. Chem. 278 (44), 42733–42736. 10.1074/jbc.C300389200 12963728

[B23] Campi M. D'Andrea L. Emiliani J. Casati P. (2012). Participation of chromatin-remodeling proteins in the repair of ultraviolet-B-damaged DNA. Plant Physiol. 158 (2), 981–995. 10.1104/pp.111.191452 22170978 PMC3271783

[B24] Cao T. Sun L. Jiang Y. Huang S. Wang J. Chen Z. (2016). Crystal structure of a nuclear actin ternary complex. Proc. Natl. Acad. Sci. U.S.A. 113 (32), 8985–8990. 10.1073/pnas.1602818113 27457955 PMC4987789

[B25] Carrozza M. J. Li B. Florens L. Suganuma T. Swanson S. K. Lee K. K. (2005). Histone H3 methylation by Set2 directs deacetylation of coding regions by Rpd3S to suppress spurious intragenic transcription. Cell 123 (4), 581–592. 10.1016/j.cell.2005.10.023 16286007

[B26] Chen L. Conaway R. C. Conaway J. W. (2013). Multiple modes of regulation of the human Ino80 SNF2 ATPase by subunits of the INO80 chromatin-remodeling complex. Proc. Natl. Acad. Sci. U. S. A. 110 (51), 20497–20502. 10.1073/pnas.1317092110 24297934 PMC3870706

[B27] Cheng X. Côté J. (2014). A new companion of elongating RNA Polymerase II: TINTIN, an independent sub-module of NuA4/TIP60 for nucleosome transactions. Transcription 5 (5), e995571. 10.1080/21541264.2014.995571 25514756 PMC4581353

[B28] Cheung A. C. M. Díaz-Santín L. M. (2019). Share and share alike: the role of Tra1 from the SAGA and NuA4 coactivator complexes. Transcription 10 (1), 37–43. 10.1080/21541264.2018.1530936 30375921 PMC6351133

[B29] Chittuluru J. R. Chaban Y. Monnet-Saksouk J. Carrozza M. J. Sapountzi V. Selleck W. (2011). Structure and nucleosome interaction of the yeast NuA4 and Piccolo-NuA4 histone acetyltransferase complexes. Nat. Struct. Mol. Biol. 18 (11), 1196–1203. 10.1038/nsmb.2128 21984211 PMC3210417

[B30] Choi K. Kim S. Kim S. Y. Kim M. Hyun Y. Lee H. (2005). SUPPRESSOR OF FRIGIDA3 encodes a nuclear ACTIN-RELATED PROTEIN6 required for floral repression in Arabidopsis. Plant Cell 17 (10), 2647–2660. 10.1105/tpc.105.035485 16155178 PMC1242263

[B31] Choi K. Park C. Lee J. Oh M. Noh B. Lee I. (2007). Arabidopsis homologs of components of the SWR1 complex regulate flowering and plant development. Development 134 (10), 1931–1941. 10.1242/dev.001891 17470967

[B32] Choi K. Kim J. Hwang H. J. Kim S. Park C. Kim S. Y. (2011). The FRIGIDA complex activates transcription of FLC, a strong flowering repressor in Arabidopsis, by recruiting chromatin modification factors. Plant Cell 23 (1), 289–303. 10.1105/tpc.110.075911 21282526 PMC3051252

[B33] Cigliano R. A. Sanseverino W. Cremona G. Ercolano M. R. Conicella C. Consiglio F. M. (2013). Genome-wide analysis of histone modifiers in tomato: gaining an insight into their developmental roles. BMC Genomics 14, 57. 10.1186/1471-2164-14-57 23356725 PMC3567966

[B34] Clapier C. R. Iwasa J. Cairns B. R. Peterson C. L. (2017). Mechanisms of action and regulation of ATP-dependent chromatin-remodelling complexes. Nat. Rev. Mol. Cell Biol. 18 (7), 407–422. 10.1038/nrm.2017.26 28512350 PMC8127953

[B35] Clarke A. S. Lowell J. E. Jacobson S. J. Pillus L. (1999). Esa1p is an essential histone acetyltransferase required for cell cycle progression. Mol. Cell Biol. 19 (4), 2515–2526. 10.1128/mcb.19.4.2515 10082517 PMC84044

[B36] Coleman-Derr D. Zilberman D. (2012). Deposition of histone variant H2A.Z within gene bodies regulates responsive genes. PloS Genet. 8 (10), e1002988. 10.1371/journal.pgen.1002988 23071449 PMC3469445

[B37] Crevillén P. Dean C. (2011). Regulation of the floral repressor gene FLC: the complexity of transcription in a chromatin context. Curr. Opin. Plant Biol. 14 (1), 38–44. 10.1016/j.pbi.2010.08.015 20884277

[B38] Crevillén P. Gómez-Zambrano A. López J. A. Vázquez J. Piñeiro M. Jarillo J. A. (2019). Arabidopsis YAF9 histone readers modulate flowering time through NuA4-complex-dependent H4 and H2A.Z histone acetylation at FLC chromatin. New Phytol. 222 (4), 1893–1908. 10.1111/nph.15737 30742710

[B39] Deal R. B. Kandasamy M. K. McKinney E. C. Meagher R. B. (2005). The nuclear actin-related protein ARP6 is a pleiotropic developmental regulator required for the maintenance of FLOWERING LOCUS C expression and repression of flowering in Arabidopsis. Plant Cell 17 (10), 2633–2646. 10.1105/tpc.105.035196 16141450 PMC1242262

[B40] Deal R. B. Topp C. N. McKinney E. C. Meagher R. B. (2007). Repression of flowering in Arabidopsis requires activation of FLOWERING LOCUS C expression by the histone variant H2A.Z. Plant Cell 19 (1), 74–83. 10.1105/tpc.106.048447 17220196 PMC1820970

[B41] Doyon Y. Côté J. (2004). The highly conserved and multifunctional NuA4 HAT complex. Curr. Opin. Genet. Dev. 14 (2), 147–154. 10.1016/j.gde.2004.02.009 15196461

[B42] Doyon Y. Selleck W. Lane W. S. Tan S. Côté J. (2004). Structural and functional conservation of the NuA4 histone acetyltransferase complex from yeast to humans. Mol. Cell Biol. 24 (5), 1884–1896. 10.1128/mcb.24.5.1884-1896.2004 14966270 PMC350560

[B43] Earley K. W. Shook M. S. Brower-Toland B. Hicks L. Pikaard C. S. (2007). In vitro specificities of Arabidopsis co-activator histone acetyltransferases: implications for histone hyperacetylation in gene activation. Plant J. 52 (4), 615–626. 10.1111/j.1365-313X.2007.03264.x 17877703

[B44] Eisen A. Utley R. T. Nourani A. Allard S. Schmidt P. Lane W. S. (2001). The yeast NuA4 and Drosophila MSL complexes contain homologous subunits important for transcription regulation. J. Biol. Chem. 276 (5), 3484–3491. 10.1074/jbc.M008159200 11036083

[B45] Finn R. D. Clements J. Eddy S. R. (2011). HMMER web server: interactive sequence similarity searching. Nucleic Acids Res. 39, W29–W37. 10.1093/nar/gkr367 21593126 PMC3125773

[B46] Gómez-Zambrano A. Crevillén P. Franco-Zorrilla J. M. López J. A. Moreno-Romero J. Roszak P. (2018). Arabidopsis SWC4 binds DNA and recruits the SWR1 complex to modulate histone H2A.Z deposition at key regulatory genes. Mol. Plant 11 (6), 815–832. 10.1016/j.molp.2018.03.014 29604400

[B47] Gerhold C. B. Gasser S. M. (2014). INO80 and SWR complexes: relating structure to function in chromatin remodeling. Trends. Cell Biol. 24 (11), 619–631. 10.1016/j.tcb.2014.06.004 25088669

[B48] Ginsburg D. S. Govind C. K. Hinnebusch A. G. (2009). NuA4 lysine acetyltransferase Esa1 is targeted to coding regions and stimulates transcription elongation with Gcn5. Mol. Cell Biol. 29 (24), 6473–6487. 10.1128/MCB.01033-09 19822662 PMC2786879

[B49] Guérillon C. Larrieu D. Pedeux R. (2013). ING1 and ING2: multifaceted tumor suppressor genes. Cell Mol. Life Sci. 70 (20), 3753–3772. 10.1007/s00018-013-1270-z 23412501 PMC11113716

[B50] He G. H. Helbing C. C. Wagner M. J. Sensen C. W. Riabowol K. (2005). Phylogenetic analysis of the ING family of PHD finger proteins. Mol. Biol. Evol. 22 (1), 104–116. 10.1093/molbev/msh256 15356280

[B51] He Y. (2012). Chromatin regulation of flowering. Trends Plant Sci. 17 (9), 556–562. 10.1016/j.tplants.2012.05.001 22658650

[B52] Helmlinger D. Tora L. (2017). Sharing the SAGA. Trends Biochem. Sci. 42 (11), 850–861. 10.1016/j.tibs.2017.09.001 28964624 PMC5660625

[B53] Helmlinger D. Marguerat S. Villén J. Swaney D. L. Gygi S. P. Bähler J. (2011). Tra1 has specific regulatory roles, rather than global functions, within the SAGA co-activator complex. EMBO J. 30 (14), 2843–2852. 10.1038/emboj.2011.181 21642955 PMC3160243

[B54] Hirayama T. Umezawa T. (2010). The PP2C-SnRK2 complex: the central regulator of an abscisic acid signaling pathway. Plant Signal Behav. 5 (2), 160–163. 10.4161/psb.5.2.10460 20023393 PMC2884124

[B55] Hodges A. J. Plummer D. A. Wyrick J. J. (2019). NuA4 acetyltransferase is required for efficient nucleotide excision repair in yeast. DNA Repair (Amst) 73, 91–98. 10.1016/j.dnarep.2018.11.006 30473425 PMC6312484

[B56] House N. C. Koch M. R. Freudenreich C. H. (2014). Chromatin modifications and DNA repair: beyond double-strand breaks. Front. Genet. 5, 296. 10.3389/fgene.2014.00296 25250043 PMC4155812

[B57] Hu Y. Lu Y. Zhao Y. Zhou D. X. (2019). Histone acetylation dynamics integrates metabolic activity to regulate plant response to stress. Front. Plant Sci. 10, 1236. 10.3389/fpls.2019.01236 31636650 PMC6788390

[B58] Ito S. Kayukawa N. Ueda T. Taniguchi H. Morioka Y. Hongo F. (2018). MRGBP promotes AR-mediated transactivation of KLK3 and TMPRSS2 *via* acetylation of histone H2A.Z in prostate cancer cells. Biochim. Biophys. Acta Gene Regul. Mech. 1861 (9), 794–802. 10.1016/j.bbagrm.2018.07.014 30076933

[B59] Jarillo J. A. Piñeiro M. (2015). H2A.Z mediates different aspects of chromatin function and modulates flowering responses in Arabidopsis. Plant J. 83 (1), 96–109. 10.1111/tpj.12873 25943140

[B60] Jin J. Shi J. Liu B. Liu Y. Huang Y. Yu Y. (2015). MORF-RELATED GENE702, a reader protein of trimethylated histone H3 lysine 4 and histone H3 lysine 36, is involved in brassinosteroid-regulated growth and flowering time control in rice. Plant Physiol. 168 (4), 1275–1285. 10.1104/pp.114.255737 25855537 PMC4528726

[B61] Kandasamy M. K. McKinney E. C. Meagher R. B. (2003). Cell cycle-dependent association of Arabidopsis actin-related proteins AtARP4 and AtARP7 with the nucleus. Plant J. 33 (5), 939–948. 10.1046/j.1365-313x.2003.01691.x 12609034

[B62] Kandasamy M. K. Deal R. B. McKinney E. C. Meagher R. B. (2005). Silencing the nuclear actin-related protein AtARP4 in Arabidopsis has multiple effects on plant development, including early flowering and delayed floral senescence. Plant J. 41 (6), 845–858. 10.1111/j.1365-313X.2005.02345.x 15743449

[B63] Keogh M. C. Kurdistani S. K. Morris S. A. Ahn S. H. Podolny V. Collins S. R. (2005). Cotranscriptional set2 methylation of histone H3 lysine 36 recruits a repressive Rpd3 complex. Cell 123 (4), 593–605. 10.1016/j.cell.2005.10.025 16286008

[B64] Keogh M. C. Mennella T. A. Sawa C. Berthelet S. Krogan N. J. Wolek A. (2006). The *Saccharomyces cerevisiae* histone H2A variant Htz1 is acetylated by NuA4. Genes Dev. 20 (6), 660–665. 10.1101/gad.1388106 16543219 PMC1413285

[B65] Klein B. J. Ahmad S. Vann K. R. Andrews F. H. Mayo Z. A. Bourriquen G. (2018). Yaf9 subunit of the NuA4 and SWR1 complexes targets histone H3K27ac through its YEATS domain. Nucleic Acids Res. 46 (1), 421–430. 10.1093/nar/gkx1151 29145630 PMC5758897

[B66] Kouzarides T. (2007). Chromatin modifications and their function. Cell 128 (4), 693–705. 10.1016/j.cell.2007.02.005 17320507

[B67] Kumar S. V. Lucyshyn D. Jaeger K. E. Alos E. Alvey E. Harberd N. P. (2012). Transcription factor PIF4 controls the thermosensory activation of flowering. Nature 484 (7393), 242–245. 10.1038/nature10928 22437497 PMC4972390

[B68] Lázaro A. Gómez-Zambrano A. López-González L. Piñeiro M. Jarillo J. A. (2008). Mutations in the Arabidopsis SWC6 gene, encoding a component of the SWR1 chromatin remodelling complex, accelerate flowering time and alter leaf and flower development. J. Exp. Bot. 59 (3), 653–666. 10.1093/jxb/erm332 18296430

[B69] Laloum T. Martin G. Duque P. (2018). Alternative Splicing Control of Abiotic Stress Responses. Trends Plant Sci. 23 (2), 140–150. 10.1016/j.tplants.2017.09.019 29074233

[B70] Latrasse D. Benhamed M. Henry Y. Domenichini S. Kim W. Zhou D. X. (2008). The MYST histone acetyltransferases are essential for gametophyte development in Arabidopsis. BMC Plant Biol. 8, 121. 10.1186/1471-2229-8-121 19040736 PMC2606689

[B71] Law J. A. Jacobsen S. E. (2010). Establishing, maintaining and modifying DNA methylation patterns in plants and animals. Nat. Rev. Genet. 11 (3), 204–220. 10.1038/nrg2719 20142834 PMC3034103

[B72] Lee K. K. Workman J. L. (2007). Histone acetyltransferase complexes: one size doesn't fit all. Nat. Rev. Mol. Cell Biol. 8 (4), 284–295. 10.1038/nrm2145 17380162

[B73] Lee W. Y. Lee D. Chung W. I. Kwon C. S. (2009). Arabidopsis ING and Alfin1-like protein families localize to the nucleus and bind to H3K4me3/2 *via* plant homeodomain fingers. Plant J. 58 (3), 511–524. 10.1111/j.1365-313X.2009.03795.x 19154204

[B74] Letunic I. Bork P. (2018). 20 years of the SMART protein domain annotation resource. Nucleic Acids Res. 46 (D1), D493–D496. 10.1093/nar/gkx922 29040681 PMC5753352

[B75] Li L. Ljung K. Breton G. Schmitz R. J. Pruneda-Paz J. Cowing-Zitron C. (2012). Linking photoreceptor excitation to changes in plant architecture. Genes Dev. 26 (8), 785–790. 10.1101/gad.187849.112 22508725 PMC3337452

[B76] Li Z. Jiang D. He Y. (2018). FRIGIDA establishes a local chromosomal environment for FLOWERING LOCUS C mRNA production. Nat. Plants 4 (10), 836–846. 10.1038/s41477-018-0250-6 30224662

[B77] Lin Y. Y. Lu J. Y. Zhang J. Walter W. Dang W. Wan J. (2009). Protein acetylation microarray reveals that NuA4 controls key metabolic target regulating gluconeogenesis. Cell 136 (6), 1073–1084. 10.1016/j.cell.2009.01.033 19303850 PMC2696288

[B78] Lin C. L. Chaban Y. Rees D. M. McCormack E. A. Ocloo L. Wigley D. B. (2017). Functional characterization and architecture of recombinant yeast SWR1 histone exchange complex. Nucleic Acids Res. 45 (12), 7249–7260. 10.1093/nar/gkx414 28499038 PMC5499540

[B79] Liu X. Yang S. Zhao M. Luo M. Yu C. W. Chen C. Y. (2014). Transcriptional repression by histone deacetylases in plants. Mol. Plant 7 (5), 764–772. 10.1093/mp/ssu033 24658416

[B80] Liu X. Yang S. Yu C. W. Chen C. Y. Wu K. (2016). Histone acetylation and plant development. Enzymes 40, 173–199. 10.1016/bs.enz.2016.08.001 27776781

[B81] Lu P. Y. Lévesque N. Kobor M. S. (2009). NuA4 and SWR1-C: two chromatin-modifying complexes with overlapping functions and components. Biochem. Cell Biol. 87 (5), 799–815. 10.1139/O09-062 19898529

[B82] Luger K. Mäder A. W. Richmond R. K. Sargent D. F. Richmond T. J. (1997). Crystal structure of the nucleosome core particle at 2.8 Å resolution. Nature 389 (6648), 251–260. 10.1038/38444 9305837

[B83] Luger K. Dechassa M. L. Tremethick D. J. (2012). New insights into nucleosome and chromatin structure: an ordered state or a disordered affair? Nat. Rev. Mol. Cell Biol. 13 (7), 436–447. 10.1038/nrm3382 22722606 PMC3408961

[B84] Martín-Trillo M. Lázaro A. Poethig R. S. Gómez-Mena C. Piñeiro M. A. Martínez-Zapater J. M. (2006). EARLY IN SHORT DAYS 1 (ESD1) encodes ACTIN-RELATED PROTEIN 6 (AtARP6), a putative component of chromatin remodelling complexes that positively regulates FLC accumulation in Arabidopsis. Development 133 (7), 1241–1252. 10.1242/dev.02301 16495307

[B85] Meagher R. B. Deal R. B. Kandasamy M. K. McKinney E. C. (2005). Nuclear actin-related proteins as epigenetic regulators of development. Plant Physiol. 139 (4), 1576–1585. 10.1104/pp.105.072447 16339804 PMC1310543

[B86] Mehta M. Braberg H. Wang S. Lozsa A. Shales M. Solache A. (2010). Individual lysine acetylations on the N terminus of *Saccharomyces cerevisiae* H2A.Z are highly but not differentially regulated. J. Biol. Chem. 285 (51), 39855–39865. 10.1074/jbc.M110.185967 20952395 PMC3000967

[B87] Millar C. B. Xu F. Zhang K. Grunstein M. (2006). Acetylation of H2A.Z Lys 14 is associated with genome-wide gene activity in yeast. Genes Dev. 20 (6), 711–722. 10.1101/gad.1395506 16543223 PMC1413291

[B88] Mitchell L. Lambert J. P. Gerdes M. Al-Madhoun A. S. Skerjanc I. S. Figeys D. (2008). Functional dissection of the NuA4 histone acetyltransferase reveals its role as a genetic hub and that Eaf1 is essential for complex integrity. Mol. Cell Biol. 28 (7), 2244–2256. 10.1128/MCB.01653-07 18212056 PMC2268438

[B89] Molina O. Vargiu G. Abad M. A. Zhiteneva A. Jeyaprakash A. A. Masumoto H. (2016). Epigenetic engineering reveals a balance between histone modifications and transcription in kinetochore maintenance. Nat. Commun. 7, 13334. 10.1038/ncomms13334 27841270 PMC5114538

[B90] Mouriz A. López-Gonzalez L. Jarillo J. A. Piñeiro M. (2015). PHDs govern plant development. Plant Signal Behav. 10 (7), e993253. 10.4161/15592324.2014.993253 26156103 PMC4622442

[B91] Narita T. Weinert B. T. Choudhary C. (2019). Functions and mechanisms of non-histone protein acetylation. Nat. Rev. Mol. Cell Biol. 20 (3), 156–174. 10.1038/s41580-018-0081-3 30467427

[B92] Noh Y. S. Amasino R. M. (2003). PIE1, an ISWI family gene, is required for FLC activation and floral repression in Arabidopsis. Plant Cell 15 (7), 1671–1682. 10.1105/tpc.012161 12837955 PMC165409

[B93] Olave I. A. Reck-Peterson S. L. Crabtree G. R. (2002). Nuclear actin and actin-related proteins in chromatin remodeling. Annu. Rev. Biochem. 71, 755–781. 10.1146/annurev.biochem.71.110601.135507 12045110

[B94] Pajoro A. Severing E. Angenent G. C. Immink R. G. H. (2017). Histone H3 lysine 36 methylation affects temperature-induced alternative splicing and flowering in plants. Genome Biol. 18 (1), 102. 10.1186/s13059-017-1235-x 28566089 PMC5452352

[B95] Peña P. V. Davrazou F. Shi X. Walter K. L. Verkhusha V. V. Gozani O. (2006). Molecular mechanism of histone H3K4me3 recognition by plant homeodomain of ING2. Nature 442 (7098), 100–103. 10.1038/nature04814 16728977 PMC3190580

[B96] Peng M. Li Z. Zhou N. Ma M. Jiang Y. Dong A. (2018). Linking PHYTOCHROME-INTERACTING FACTOR to Histone Modification in Plant Shade Avoidance. Plant Physiol. 176 (2), 1341–1351. 10.1104/pp.17.01189 29187567 PMC5813548

[B97] Perrella G. Carr C. Asensi-Fabado M. A. Donald N. A. Paldi K. Hannah M. A. (2016). The histone deacetylase complex 1 protein of Arabidopsis has the capacity to interact with multiple proteins including histone 3-binding proteins and histone 1 variants. Plant Physiol. 171 (1), 62–70. 10.1104/pp.15.01760 26951436 PMC4854681

[B98] Perry J. (2006). The Epc-N domain: a predicted protein-protein interaction domain found in select chromatin associated proteins. BMC Genomics 7, 6. 10.1186/1471-2164-7-6 16412250 PMC1388200

[B99] Pokholok D. K. Harbison C. T. Levine S. Cole M. Hannett N. M. Lee T. I. (2005). Genome-wide map of nucleosome acetylation and methylation in yeast. Cell 122 (4), 517–527. 10.1016/j.cell.2005.06.026 16122420

[B100] Potok M. E. Wang Y. Xu L. Zhong Z. Liu W. Feng S. (2019). Arabidopsis SWR1-associated protein methyl-CpG-binding domain 9 is required for histone H2A.Z deposition. Nat. Commun. 10 (1), 3352. 10.1038/s41467-019-11291-w 31350403 PMC6659704

[B101] Reid J. L. Moqtaderi Z. Struhl K. (2004). Eaf3 regulates the global pattern of histone acetylation in *Saccharomyces cerevisiae* . Mol. Cell Biol. 24 (2), 757–764. 10.1128/mcb.24.2.757-764.2004 14701747 PMC343795

[B102] Rossetto D. Cramet M. Wang A. Y. Steunou A. L. Lacoste N. Schulze J. M. (2014). Eaf5/7/3 form a functionally independent NuA4 submodule linked to RNA polymerase II-coupled nucleosome recycling. EMBO J. 33 (12), 1397–1415. 10.15252/embj.201386433 24843044 PMC4194127

[B103] Rothbart S. B. Strahl B. D. (2014). Interpreting the language of histone and DNA modifications. Biochim. Biophys. Acta 1839 (8), 627–643. 10.1016/j.bbagrm.2014.03.001 24631868 PMC4099259

[B104] Rountree M. R. Bachman K. E. Baylin S. B. (2000). DNMT1 binds HDAC2 and a new co-repressor, DMAP1, to form a complex at replication foci. Nat. Genet. 25 (3), 269–277. 10.1038/77023 10888872

[B105] Sapountzi V. Logan I. R. Robson C. N. (2006). Cellular functions of TIP60. Int. J. Biochem. Cell Biol. 38 (9), 1496–1509. 10.1016/j.biocel.2006.03.003 16698308

[B106] Sathianathan A. Ravichandran P. Lippi J. M. Cohen L. Messina A. Shaju S. (2016). The Eaf3/5/7 Subcomplex Stimulates NuA4 Interaction with Methylated Histone H3 Lys-36 and RNA Polymerase II. J. Biol. Chem. 291 (40), 21195–21207. 10.1074/jbc.M116.718742 27535225 PMC5076527

[B107] Schuettengruber B. Bourbon H. M. Di Croce L. Cavalli G. (2017). Genome regulation by polycomb and trithorax: 70 years and counting. Cell 171 (1), 34–57. 10.1016/j.cell.2017.08.002 28938122

[B108] Searle N. E. Pillus L. (2018). Critical genomic regulation mediated by Enhancer of Polycomb. Curr. Genet. 64 (1), 147–154. 10.1007/s00294-017-0742-3 28884217 PMC6287951

[B109] Setiaputra D. Ahmad S. Dalwadi U. Steunou A. L. Lu S. Ross J. D. (2018). Molecular architecture of the essential yeast histone acetyltransferase complex NuA4 redefines its multimodularity. Mol. Cell Biol. 38 (9), e00570-17. 10.1128/MCB.00570-17 29463645 PMC5902594

[B110] Shahbazian M. D. Grunstein M. (2007). Functions of site-specific histone acetylation and deacetylation. Annu. Rev. Biochem. 76, 75–100. 10.1146/annurev.biochem.76.052705.162114 17362198

[B111] Sijacic P. Holder D. H. Bajic M. Deal R. B. (2019). Methyl-CpG-binding domain 9 (MBD9) is required for H2A.Z incorporation into chromatin at a subset of H2A.Z-enriched regions in the Arabidopsis genome. PloS Genet. 15 (8), e1008326. 10.1371/journal.pgen.1008326 31381567 PMC6695207

[B112] Song Y. H. Shim J. S. Kinmonth-Schultz H. A. Imaizumi T. (2015). Photoperiodic flowering: time measurement mechanisms in leaves. Annu. Rev. Plant Biol. 66, 441–464. 10.1146/annurev-arplant-043014-115555 25534513 PMC4414745

[B113] Springer N. M. Danilevskaya O. N. Hermon P. Helentjaris T. G. Phillips R. L. Kaeppler H. F. (2002). Sequence relationships, conserved domains, and expression patterns for maize homologs of the polycomb group genes E(z), esc, and E(Pc). Plant Physiol. 128 (4), 1332–1345. 10.1104/pp.010742 11950982 PMC154261

[B114] Steunou A. L. Cramet M. Rossetto D. Aristizabal M. J. Lacoste N. Drouin S. (2016). Combined action of histone reader modules regulates NuA4 local acetyltransferase function but not its recruitment on the genome. Mol. Cell Biol. 36 (22), 2768–2781. 10.1128/MCB.00112-16 27550811 PMC5086519

[B115] Strahl B. D. Allis C. D. (2000). The language of covalent histone modifications. Nature 403 (6765), 41–45. 10.1038/47412 10638745

[B116] Su Y. Wang S. Zhang F. Zheng H. Liu Y. Huang T. (2017). Phosphorylation of histone H2A at serine 95: a plant-specific mark involved in flowering time regulation and H2A.Z deposition. Plant Cell 29 (9), 2197–2213. 10.1105/tpc.17.00266 28790150 PMC5635989

[B117] Szerlong H. Hinata K. Viswanathan R. Erdjument-Bromage H. Tempst P. Cairns B. R. (2008). The HSA domain binds nuclear actin-related proteins to regulate chromatin-remodeling ATPases. Nat. Struct. Mol. Biol. 15 (5), 469–476. 10.1038/nsmb.1403 18408732 PMC2810487

[B118] Talbert P. B. Henikoff S. (2017). Histone variants on the move: substrates for chromatin dynamics. Nat. Rev. Mol. Cell Biol. 18 (2), 115–126. 10.1038/nrm.2016.148 27924075

[B119] Tan L. M. Zhang C. J. Hou X. M. Shao C. R. Lu Y. J. Zhou J. X. (2018). The PEAT protein complexes are required for histone deacetylation and heterochromatin silencing. EMBO J. 37 (19), e98770. 10.15252/embj.201798770 30104406 PMC6166130

[B120] Taverna S. D. Ilin S. Rogers R. S. Tanny J. C. Lavender H. Li H. (2006). Yng1 PHD finger binding to H3 trimethylated at K4 promotes NuA3 HAT activity at K14 of H3 and transcription at a subset of targeted ORFs. Mol. Cell 24 (5), 785–796. 10.1016/j.molcel.2006.10.026 17157260 PMC4690528

[B121] Umezawa T. Sugiyama N. Takahashi F. Anderson J. C. Ishihama Y. Peck S. C. (2013). Genetics and phosphoproteomics reveal a protein phosphorylation network in the abscisic acid signaling pathway in *Arabidopsis thaliana* . Sci. Signal 6 (270), rs8. 10.1126/scisignal.2003509 23572148

[B122] Uprety B. Lahudkar S. Malik S. Bhaumik S. R. (2012). The 19S proteasome subcomplex promotes the targeting of NuA4 HAT to the promoters of ribosomal protein genes to facilitate the recruitment of TFIID for transcriptional initiation *in vivo* . Nucleic Acids Res. 40 (5), 1969–1983. 10.1093/nar/gkr977 22086954 PMC3300024

[B123] Uprety B. Sen R. Bhaumik S. R. (2015). Eaf1p is required for recruitment of NuA4 in targeting TFIID to the promoters of the ribosomal protein genes for transcriptional initiation *in vivo* . Mol. Cell Biol. 35 (17), 2947–2964. 10.1128/MCB.01524-14 26100014 PMC4525319

[B124] Valdés-Mora F. Song J. Z. Statham A. L. Strbenac D. Robinson M. D. Nair S. S. (2012). Acetylation of H2A.Z is a key epigenetic modification associated with gene deregulation and epigenetic remodeling in cancer. Genome Res. 22 (2), 307–321. 10.1101/gr.118919.110 21788347 PMC3266038

[B125] Venkatesh S. Workman J. L. (2015). Histone exchange, chromatin structure and the regulation of transcription. Nat. Rev. Mol. Cell Biol. 16 (3), 178–189. 10.1038/nrm3941 25650798

[B126] Voss A. K. Thomas T. (2009). MYST family histone acetyltransferases take center stage in stem cells and development. Bioessays 31 (10), 1050–1061. 10.1002/bies.200900051 19722182

[B127] Wang A. Y. Schulze J. M. Skordalakes E. Gin J. W. Berger J. M. Rine J. (2009). Asf1-like structure of the conserved Yaf9 YEATS domain and role in H2A.Z deposition and acetylation. Proc. Natl. Acad. Sci. U.S.A. 106 (51), 21573–21578. 10.1073/pnas.0906539106 19966225 PMC2799888

[B128] Wang X. Ahmad S. Zhang Z. Côté J. Cai G. (2018a). Architecture of the *Saccharomyces cerevisiae* NuA4/TIP60 complex. Nat. Commun. 9 (1), 1147. 10.1038/s41467-018-03504-5 29559617 PMC5861120

[B129] Wang X. Zhu W. Chang P. Wu H. Liu H. Chen J. (2018b). Merge and separation of NuA4 and SWR1 complexes control cell fate plasticity in *Candida albicans* . Cell Discovery 4, 45. 10.1038/s41421-018-0043-0 30109121 PMC6089883

[B130] Xiao J. Zhang H. Xing L. Xu S. Liu H. Chong K. (2013). Requirement of histone acetyltransferases HAM1 and HAM2 for epigenetic modification of FLC in regulating flowering in Arabidopsis. J. Plant Physiol. 170 (4), 444–451. 10.1016/j.jplph.2012.11.007 23273925

[B131] Xiao J. Jin R. Wagner D. (2017). Developmental transitions: integrating environmental cues with hormonal signaling in the chromatin landscape in plants. Genome Biol. 18 (1), 88. 10.1186/s13059-017-1228-9 28490341 PMC5425979

[B132] Xie T. Zmyslowski A. M. Zhang Y. Radhakrishnan I. (2015). Structural basis for multi-specificity of MRG domains. Structure 23 (6), 1049–1057. 10.1016/j.str.2015.03.020 25960410 PMC4456287

[B133] Xu Y. Gan E. S. Zhou J. Wee W. Y. Zhang X. Ito T. (2014). Arabidopsis MRG domain proteins bridge two histone modifications to elevate expression of flowering genes. Nucleic Acids Res. 42 (17), 10960–10974. 10.1093/nar/gku781 25183522 PMC4176166

[B134] Xu P. Li C. Chen Z. Jiang S. Fan S. Wang J. (2016). The NuA4 core complex acetylates nucleosomal histone H4 through a double recognition mechanism. Mol. Cell 63 (6), 965–975. 10.1016/j.molcel.2016.07.024 27594449

[B135] Yang X. J. Ullah M. (2007). MOZ and MORF, two large MYSTic HATs in normal and cancer stem cells. Oncogene 26 (37), 5408–5419. 10.1038/sj.onc.1210609 17694082

[B136] Yi C. Ma M. Ran L. Zheng J. Tong J. Zhu J. (2012). Function and molecular mechanism of acetylation in autophagy regulation. Science 336 (6080), 474–477. 10.1126/science.1216990 22539722

[B137] Zacharaki V. Benhamed M. Poulios S. Latrasse D. Papoutsoglou P. Delarue M. (2012). The Arabidopsis ortholog of the YEATS domain containing protein YAF9a regulates flowering by controlling H4 acetylation levels at the FLC locus. Plant Sci. 196, 44–52. 10.1016/j.plantsci.2012.07.010 23017898

[B138] Zhou B. O. Wang S. S. Xu L. X. Meng F. L. Xuan Y. J. Duan Y. M. (2010). SWR1 complex poises heterochromatin boundaries for antisilencing activity propagation. Mol. Cell Biol. 30 (10), 2391–2400. 10.1128/MCB.01106-09 20308321 PMC2863710

